# The giant pill-millipede genus *Zephronia* Gray, 1832 from Thailand, with a redescription of *Z.siamensis* Hirst, 1907 and descriptions of three new species (Diplopoda, Sphaerotheriida, Zephroniidae)

**DOI:** 10.3897/zookeys.1067.72369

**Published:** 2021-10-29

**Authors:** Ruttapon Srisonchai, Chirasak Sutcharit, Natdanai Likhitrakarn

**Affiliations:** 1 Department of Biology, Faculty of Science, Khon Kaen University, Khon Kaen, 40002, Thailand; 2 Animal Systematics Research Unit, Department of Biology, Faculty of Science, Chulalongkorn University, Bangkok, 10330, Thailand; 3 Division of Plant Protection, Faculty of Agricultural Production, Maejo University, Chiang Mai, 50290, Thailand; 4 Biodiversity and Utilization Research Center of Maejo University, Maejo University, Chiang Mai, 50290, Thailand

**Keywords:** Diplopods, key, map, Southeast Asia, taxonomy

## Abstract

Material of the giant pill-millipede genus *Zephronia* Gray, 1832 recently collected from Thailand contains three new species: *Zephroniaenghoffi***sp. nov.**, *Zephroniagolovatchi***sp. nov.**, and *Zephroniapanhai***sp. nov.** The first *Zephronia* species recorded for Thailand, *Z.siamensis* Hirst, 1907, is also redescribed based on new specimens collected both from the type locality in Chonburi Province and from neighboring areas. Morphological characters of all new species, *Z.phrain* Likhitrakarn & Golovatch, 2021, and *Z.siamensis* are illustrated, and a distribution map of the confirmed *Zephronia* species occurring in Thailand is also provided.

## Introduction

One of the remarkable diplopod groups, the giant pill-millipede genus *Zephronia* Gray, 1832 is one of the most speciose not only in the family Zephroniidae, but also in the entire order Sphaerotheriida. It currently contains 44 described species ranging from the Himalayas of India in the west, to mainland Southeast Asia in the east (Wesener 2016, 2019). Although several species have been revised and new species described from a number of areas in Asia, e.g., Myanmar, Northeast India, and Vietnam, *Zephronia* diversity still remains understudied in many other countries, e.g., Cambodia, Laos, and Thailand. Thailand is located within one of the global hotspots of biodiversity (Indo-Burma) (Clements et al. 2006), and even though recent progress in revealing its diplopod fauna is considerable, especially as regards the orders Spirobolida, Spirostreptida, and Polydesmida. (Pimvichai et al. 2009, 2010; Likhitrakarn et al. 2011, 2014; Srisonchai et al. 2018a, b), only four species of *Zephronia* have hitherto been reported from Thailand. These are as follows: *Z.siamensis* Hirst, 1907, *Z.lannaensis* Likhitrakarn & Golovatch, 2021, *Z.phrain* Likhitrakarn & Golovatch, 2021, and *Z.viridisoma* Rosenmejer & Wesener, 2021. Recent intense collecting efforts made by Thai specialists in collaboration with the Department of National Parks, Wildlife and Plant Conservation across the country have revealed numerous interesting millipedes, especially in limestone areas. From these efforts, several new genera and numerous new species have been recorded and described (Pimvichai et al. 2018, 2020; Srisonchai et al. 2018a, b, c, d; Likhitrakarn et al. 2020, 2021). The present contribution provides descriptions of three new species of the genus *Zephronia*, as well as a redescription of *Z.siamensis* Hirst, 1907 as based both on topotypes and near-topotypes.

## Materials and methods

### Specimen collection and preservation

The millipedes were collected by active search in daytime during the field trips in Thailand. All material was collected by **ASRU** (Animal Systematics Research Unit) members. Live specimens of both sexes were photographed with a Nikon D700 camera equipped with a AF-S VR Micro-Nikkor 105 mm lens. Specimens were then euthanized based on the methods of AVMA guidelines for the euthanasia of animals (American Veterinary Medical Association 2020) with a permission of the Animal Care and Use. Most of the specimens were stored in 70% ethanol for morphological examination. Latitude, longitude, and elevation were recorded using a Garmin GPSMAP 60 CSx at the field sites, and all coordinates of the precise locations were mapped with Google Earth.

### Morphological study, description, and illustrations

All morphological characters were analyzed under a NIKON SMZ-445 stereo microscope. For Scanning Electron Microscopy (SEM), the specimens were mounted on aluminum stubs, coated with pure gold and studied using a JOEL JSM-6610LV scanning electron microscope. The descriptions are applied to both males and females. Species delimitation and morphological descriptions were based on Wesener and Sierwald (2005), Wesener (2016, 2019), Semenyuk et al. (2018, 2020) and Likhitrakarn et al. (2021). Illustrations of external morphological characters were sketched from one view, whereas the telopods were depicted from three sides (anterior, posterior, and lateral views) under the stereo microscope and all were modified using Adobe Photoshop CS6 software in order to generate plates of figures.

### Depositions of holotypes, paratypes, and other new specimens

All material of each species is referred to each species description. The holotypes are deposited in the Chulalongkorn University Museum of Zoology (**CUMZ**, CUMZ-Zeph0005-0010) and some paratypes are shared with three other museums including the Natural History Museum of Denmark, University of Copenhagen, Denmark (NHMD), the Zoological Museum, State University of Moscow, Russia (**ZMUM**), and the Zoological Reference Collection of the Lee Kong Chian Natural History Museum, Singapore (**ZRC**).

### Acronyms used in the descriptions

**cp** cuticular impression

**cr-T** crenulated teeth

**cx** coxa

**is** inner section

**ML** membranous lobe

**ms** middle section

**o** operculum of vulva

**ot** outer section

**pm** posterior margin

**pre** prefemur

**sp** sclerotized process

**st-pl** stigmatic plate

**syn-cx** syncoxite

### Other acronyms and words used in the text

**ASRU** Animal Systematics Research Unit, Chulalongkorn University, Thailand

**a.s.l.** above sea-level

**ca.** about, around, circa

**CUMZ** Chulalongkorn University Museum of Zoology, Thailand

**Koh** the Thai word for “island”

**NHMD** Natural History Museum of Denmark, University of Copenhagen, Denmark

**Wat** the Thai word for “temple”

**ZMUM** Zoological Museum, State University of Moscow, Russia.

### Positional and directional terms used in the descriptions

See the details in species descriptions by Wesener (2019), Likhitrakarn et al. (2021), and also some definitions in Srisonchai et al. (2018a, b).

## Results

### Family Zephroniidae Gray, 1843

**Subfamily Zephroniinae Gray, 1843**

**Tribe Zephroniini Jeekel, 2001**

#### Genus Zephronia

Taxon classificationAnimaliaSphaerotheriidaZephroniidae

Gray, 1832

0696B082-AB5D-5A13-B9D8-573CA561B05D

##### Diagnosis.

See complete and recently updated diagnoses in Golovatch et al. (2012: 283), Wesener (2016: 30), and Likhitrakarn et al. (2021: 13).

##### Confirmed species recorded from Thailand.

1. *Zephroniasiamensis* Hirst, 1907 2. *Zephronialannaensis* Likhitrakarn & Golovatch, 2021 3. *Zephroniaphrain* Likhitrakarn & Golovatch, 2021 4. *Zephroniaviridisoma* Rosenmejer & Wesener, 2021 5. *Zephroniaenghoffi*sp. nov. 6. *Zephroniagolovatchi*sp. nov. 7. *Zephroniapanhai*sp. nov.

##### Unconfirmed species recorded for Thailand.

Zephroniacf.viridescens Attems, 1936.

#### Zephronia siamensis

Taxon classificationAnimaliaSphaerotheriidaZephroniidae

Hirst, 1907

B86C57C0-6EFC-5888-AC19-C847D7394223

**Figure 1.**
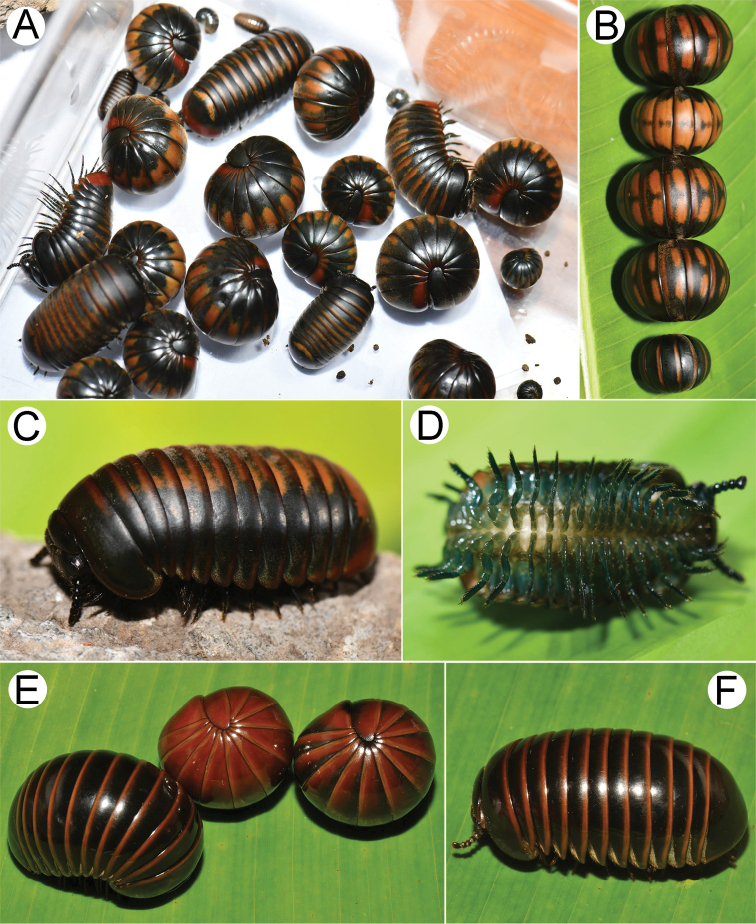
Photographs of living *Zephronia* spp. **A–D***Zephroniasiamensis***E, F***Zephroniaphrain*. Photographs not to scale.

Figures 1A–D ; 3 ; 4 ; 13A, B ; 14A

Zephronia siamensis Hirst, 1907: 218; Attems 1914: 147; Attems 1936: 182; Jeekel 2001: 21; Enghoff 2005: 89; Golovatch et al. 2012: 276; Wongthamwanich et al. 2012a: 30; Wesener 2016: 35.
Zephronia cf. siamensis – Decker 2010: 25.

##### Material examined.

Thailand – Chonburi Province • 2 ♂♂ 17 ♀♀; Sichang District, Koh Sichang; 13°9'3.8"N, 100°48'56"E; 7 m a.s.l.; 14 November 2020; R. Srisonchai, N. Likhitrakarn, P. Jirapatrasilp leg.; • 2 ♀♀; same collection data; NHMD • 2 ♀♀; same collection data; ZMUM • 3 ♀♀; same Province, Mueang District, Grand Cayon Chonburi; 12°31'23"N, 100°57'18"E; 7 m a.s.l.; 2 August 2019; ASRU members leg.; • 1 ♀; same Province, Sattahip District, Koh Chuang; 12°31'23"N, 100°57'18"E; 7 m a.s.l.; 8 August 2013; R. Srisonchai, P. Jirapatrasilp leg.; • 2 ♀♀; same Province, Bo Thong District, Wat Tham Khao Cha-ang-on; 13°12'31.7"N, 101°39'5.7"E; 128 m a.s.l.; 4 July 2016; R. Srisonchai, P. Tongkerd leg.; • 1 ♀; Rayong Province, Mueang District, Koh Samet; 12°34'22.6"N, 101°27'52.6"E; 128 m a.s.l.; 12 January 2010; ASRU members leg.

##### Type locality.

Kosichang and Chantaboon, Siam (Hirst 1907), [Koh Sichang (Island) is in Chonburi Province, Chantaboon is in Chantaburi Province].

##### Diagnosis.

A member of *Zephronia* s. s. in which the position of Tömösváry’s organ located next to the aberrant ommatidia, not inside the antennal groove. Adult body length relatively small, usually ca. 20 mm, < 26.5 mm, tip of subanal plate concave, process of telopoditomere 2 of anterior telopod rather short and strongly curved distally, and process of telopoditomere 2 of anterior telopod shorter than the combination of telopoditomeres 3 and 4. Similar in these respects to *Z.laotica* Wesener, 2019 and *Z.dawydoffi* Attems, 1953. Differs from these two species by showing a body length > 16.7 mm, live specimens with unique dark green, tergites with two yellow-brown patches located in anterior half of tergites, surface of tergites with conspicuous setae, femur of leg extended with conspicuous teeth, and telopoditomere 4 of anterior telopods posteriorly with a row of conspicuous crenulated teeth (cr-T).

##### Redescription.

***Body size***: Male: body length 15.0–26.5 mm. Width of thoracic shield 9.0–12.5 mm, of tergite 8 9.5–11.4 mm. Height of thoracic shield 5.2–6.4 mm, of tergite 7, 6.1–7.4 mm. Female: body length 15–23.0 mm. Width of thoracic shield ca. 12.1 mm, of tergite 8 ca. 12.8 mm. Height of thoracic shield ca. 7.3 mm, of tergite 7 ca. 8.2 mm.

***Color*** (Fig. 1A–D): Live specimens dark green; antennae dark brown; head, collum, thoracic shield, paratergites brown; legs bluish green. Tergites with two big patches, brown or yellowish brown, arranged in almost central part of anterior half; lateral part of tergites greenish dark, middle part of tergites brown. Anal shield with two colors contrasting each other, posterior half reddish brown, anterior half greenish dark brown. Color in alcohol after three months of preservation changed to greenish brown, head and collum dark greenish, tergites with a dark posterior margin, legs pale yellowish, distal podomeres rusty brown, antennae dark green.

***Head***: Trapezoid, anterior part of head clothed with numerous long setae, posterior part sparsely punctate; anterior margin of labrum with a single tooth. Each eye with 75–92 ommatidia. Aberrant ocellus located inside antennal groove.

***Antennae*** (Fig. 3A): Short, with rounded joints, extending posteriorly to leg-pair 2. Lengths of antennomeres: 6 > 3 > 5 = 4 > 2 = 1. Antennomere 6 densely pubescent, sensilla basiconica surrounding apical disc. Last antennomere thickened, widened apically and axe-shaped. Shape of antennae sexually dimorphic, cylindrical in female; thickened, widened apically and slightly flattened in male. Apical disc with 51–61 (males) or 49–54 apical cones (females). No sclerotized crest/ridge between antennal socket and ommatidia.

***Tömösváry’s organ***: Located separately at a small, projected brim between ommatidia and antennal socket.

***Gnathochilarium***: Ventral surface with setae, other structures typical of the order. Palpi with sensory cones arranged in clusters. Mandibles not dissected.

***Stigmatic plates*** (Fig. 3C): First stigmatic plate subtriangular, apex broadly rounded, slightly curved towards coxa 1.

***Laterotergites***: 1 and 2 with a broad and well-rounded projection.

***Collum***: With glabrous surface, sparsely setose with very long setae, except for anterior and posterior margins which are densely setose.

***Thoracic shield***: Surface with tiny setae as on tergites. Shallow grooves filled with numerous long setae, no keels.

***Tergites*** (Fig. 1A–C): Surface shining, entirely clothed with dense and tiny setae, each seta located in a tiny pit. Tip of paratergites weakly projecting posteriorly.

***Endotergum*** (Figs 13A, B, 14A): Posterior margin with lobes, ‘rectangle-wavy’ margin. Inner section (inner area) smooth, with a few setae. Middle section (middle area) with a single row of conspicuous, elliptical cuticular impressions; distance between impressions as wide as individual diameter. Bristles arranged in one row, tip of the longest bristles not extended beyond posterior margin or not reaching to posterior margin.

***Anal shield***: Sexually dimorphic, in female large and well-rounded, in male slightly more rectangular, in both sexes glabrous. Surface similar to that of tergites. Inner surface (underside) with a single, long, black locking carina half as long as width of last laterotergite.

***Legs*** (Fig. 3B): Leg-pair 1 with one ventral spine, leg-pair 2 with two or three, leg-pair 3 with 4–6 ventral spines. Leg-pairs 4–18 with eight or nine ventral spines and two or three apical ones; thereafter slightly reduced into 5–8 ventral spines. In leg 9, femur ca. 1.7×, tarsus ca. 3.2× longer than wide. Length of tarsus > femur > prefemur > coxa > tibia ≥ postfemur. All podomeres densely setose. Coxa large, with dentate ridge marginally (coxal process). Coxal process absent in leg-pairs 1 and 2. Prefemur without teeth. Femur large and stout, extended mesally, with 7–11 conspicuous teeth.

***Subanal plate*** (Fig. 3F): Large and wide, semicircular, divided by a conspicuous mesal constriction; central margin (tip) concave, wide; lateral margin slightly convex. Densely setose.

***Male sexual characters*** (Fig. 3D): Male gonopore large, covered with a single, undivided, triangular, sclerotized plate.

***Anterior telopods*** (Fig. 4A–C): First telopoditomere rectangular, slightly longer than wide. Telopoditomere 2 large, as long as telopoditomeres 3 and 4 combined. Process of telopoditomere 2 located posteriorly, but partly visible laterally in anterior view. Process of telopoditomere 2 wide, broader than telopoditomeres 3 and 4. Process of telopoditomere 2 conspicuously unciform, protruding as high as basal part of telopoditomere 4, apically with a well-rounded tip. Margin towards telopoditomere 3 with a membranous area carrying a rather small and sclerotized process (sp), apically with a rounded tip. Telopoditomere 3 slender, 1.4X longer than wide, 1.5X longer than telopoditomere 4. Telopoditomere 4 posteriorly with a row of 7 small and crenulated teeth (cr-T) with two prominent spines. All podomeres covered with long sparse setae, except for central part of telopoditomere 1 and posterior surfaces of 2–4.

***Posterior telopods*** (Fig. 4D–F): Inner horns with sharply edged tips, slightly curved caudad. Telopodite consisting of four podomeres. First podomere stout and narrow, nearly twice as wide as long. Immovable finger (process of telopoditomere 2) shorter than movable finger (consisting of telopoditomeres 3 and 4). Immovable finger stout and narrow, 1.6X longer than wide, not curved, glabrous distally. Margin towards movable finger with two massive, triangular, membranous lobes (ML). Telopoditomere 3 elongated, slightly curved, twice as long as telopoditomere 4; with a large, swollen, membranous ledge; postero-apically slightly enlarged, with a row of 11 or 12 crenulated teeth (cr-T). Telopoditomere 4 slender, twice as long as wide, slightly tapering apically; with a large, swollen, membranous ledge; with two long and sclerotized spines. Telopoditomeres 1 and 2 on both sides covered with few setae. Telopoditomere 3 at base of inner margin with a few setae, remaining parts of telopoditomeres 3 and 4 almost glabrous.

***Female sexual characters*** (Fig. 3E): Vulva large, covering ca. 2/3 coxa, located at mesal side, extending mesally to basal third of prefemur. Operculum regularly rounded, margin straight, mesal margin not protruding.

##### Distribution and habitats

(Figs 15A, 16). The newly collected specimens from the type locality were found under groups of *Pandanus* trees in a limestone area near a beach, while the other material from the Chonburi and Rayong provinces were likewise taken from limestone habitats. Currently, this species is known to occur only in eastern Thailand.

##### Remarks.

The live coloration of adults is generally dark green with two yellowish brown patches in the anterior half of tergites, this being quite unique for this species.

Almost 114 years since the original description, a redescription of *Z.siamensis* Hirst, 1907 has been made in this study based on the newly collected specimens from Koh Srichang (Srichang Island), here regarded as strict topotypes.

Considerable variation has been found in body size of the specimen described by Hirst (1907) compared to the topotypes: the type specimen was ca. 26.5 mm in length, whereas the new material we examined were within the size range of 16.7–23.5 mm.

#### Zephronia lannaensis

Taxon classificationAnimaliaSphaerotheriidaZephroniidae

Likhitrakarn & Golovatch, 2021

474FB4D3-B338-5775-91FC-19B53481C3CA

Zephronia lannaensis Likhitrakarn & Golovatch, 2021 in Likhitrakarn et al. 2021: 13.

##### Distribution and habitats.

This species has been found to occur only in Chiang Mai Province. (Thailand, Chiang Mai Province, Doi Saket District, Huai Hong Khrai Royal Development Study Centre, 445 m a.s.l., 18°52'"N, 99°13'"E). All specimens were collected from dry dipterocarp forest (Likhitrakaen et al. 2021).

##### Remarks.

Based on specimens described by Likhitrakarn et al. 2021, deposited in the CUMZ (holotype CUMZ-Zeph0001, paratypes CUMZ-Zeph0002).

#### Zephronia phrain

Taxon classificationAnimaliaSphaerotheriidaZephroniidae

Likhitrakarn & Golovatch, 2021

2680FE14-94DB-5F0E-8A81-2CC2DA652065

Figures 1E, F ; 5 ; 6

Zephronia phrain Likhitrakarn & Golovatch, 2021 in Likhitrakarn et al. 2021: 19.

##### Material examined.

Thailand – Tak Province • 2 ♂♂ 2 ♀♀; Mae Sot District, Phawor Shrine; 16°46'16.8"N, 98°41'13"E; 694 m a.s.l.; October 2016; S. Panha, R. Srisonchai, C. Sutcharit, W. Siriwut leg.

##### Description of some characters for a population in Tak Province.

***Body length***: Length in male 29.0–31.0 mm (holotype 33.5 mm), female 30.0–33.0 mm; head 5.5 mm; thoracic shield 5.5–6.0 mm; anal shield 9.5–10.5 mm.

***Body width***: Width in male 16.5 mm (holotype 18.2 mm), female 16.5–17.0 mm; head 8.0–9.0 mm; thoracic shield 15.0–16.0 mm; anal shield 14.0–15.5 mm.

***Body height***: Height in male 10.0 mm (holotype 11.2 mm), female 10.0–11.0 mm; thoracic shield 9.0–10.5 mm; tergite 9.5–11.0 mm.

***Color*** (Fig. 1E, F): Specimens in life with brown or dark brown; head, antennae and collum, thoracic shield, paratergites, anal shield and legs brown or dark brown; anterior margins of thoracic shield, of tergites and of anal shield dark brown contrasting with the posterior brown ones; setose part of thoracic shield with golden sheen. Color in alcohol after six years not changed.

***Tergites*** (Fig. 1E, F): Quite shiny; surface glabrous, with sparse, tiny, inconspicuous pits; tip of paratergite of midbody tergites curved, directed posteroventrad; anterior half of lateral margin covered with long and conspicuous setae.

***Legs*** (Fig. 5B): Leg-pairs 1 and 2 without apical spine. Leg-pair 1 with four ventral spines, leg-pair 2 with four or five ventral spines. Leg-pair 3 with seven or eight ventral spines and one or two apical spines. Leg-pair 4 with nine or ten ventral spines and two or three apical spines. Leg-pairs 5–19 with 9–11 ventral spines and 1–3 apical spines. Last two leg-pairs with eight or nine ventral spines, and one or two apical spines. In leg 9, femur ca. 1.7×, tarsus ca. 3.4× longer than wide. Length of tarsus > femur > prefemur > coxa > tibia ≥ postfemur. All podomeres densely setose. Coxa large, with dentate ridge marginally (coxal process). Coxal process absent in leg-pairs 1 and 2. Prefemur without teeth. Femur slightly extended mesally; mesal margin with very small, tiny, inconspicuous teeth.

***Subanal plate*** (Fig. 5F): Trapeziform, undivided; central margin (tip) truncate, narrow; lateral margin straight. Densely setose.

Head, antenna, Tömösváry’s organ, gnathochilarium, stigmatic plates, laterotergites, collum, thoracic shield, endotergum, anal shield, male sexual characters, anterior telopods, posterior telopods, and female sexual characters: Same as the original description in Likhitrakarn et al. 2021.

##### Distribution and habitats

(Figs 15D, 16). Currently known to occur in northern Thailand (Chiang Mai and Tak provinces) in dry dipterocarp forest and from limestone areas. Observations made at Phawor Shrine found that most specimens were seen creeping on rocks, with some hiding in leaf litter. Notably, the specimens were found in syntopy with the dragon millipede (*Nagaxytesspatula* Srisonchai, Enghoff & Panha, 2018) at the same site (Srisonchai et al. 2018b).

##### Remarks.

**Figure 2.**
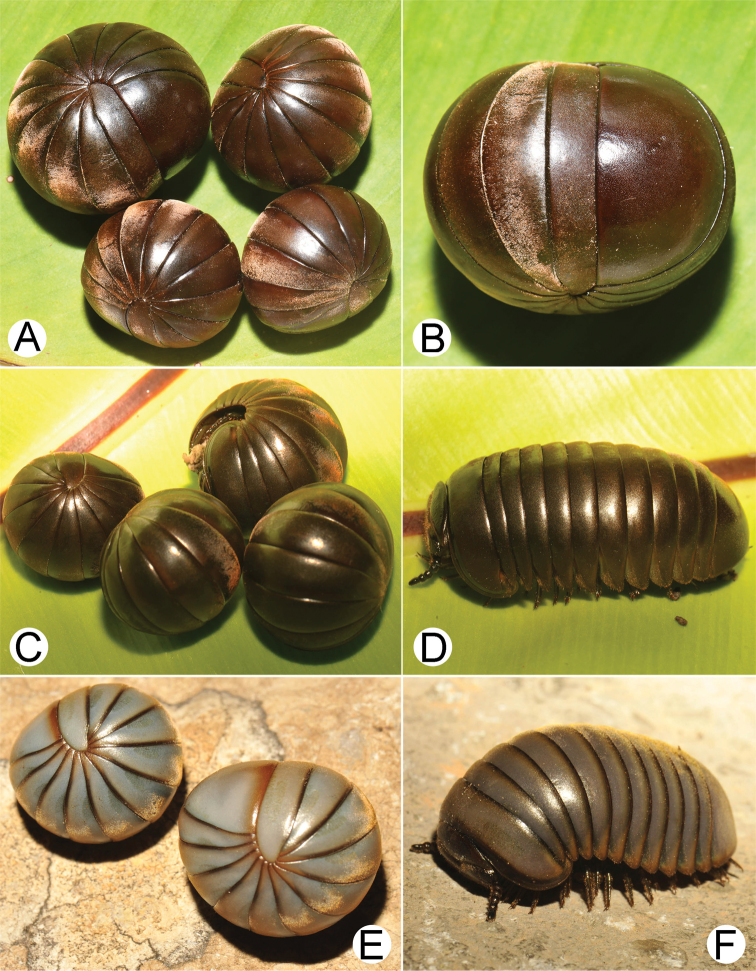
Photographs of living *Zephronia* spp. **A, B***Zephroniaenghoffi*sp. nov., paratypes (CUMZ-Zeph0006) **C, D***Zephroniagolovatchi*sp. nov., paratypes (CUMZ-Zeph0008) **E, F***Zephroniapanhai*sp. nov., paratypes (CUMZ-Zeph0010). Photographs not to scale.

Based on observations of live specimens in the field, two color patterns were found, dark green in type specimens and brown/dark brown in the others from Tak Province (Fig. 2E, F; fig. 1C, D in Likhitrakarn et al. 2021).

A species described by Pocock (1890) from Myanmar (Thagatà, Mount Mooleyit, Kayah State), *Z.gestri* Pocock, 1890 occurs close to the type locality of this widespread species, but *Z.phrain* clearly differs from *Z.gestri* by being longer in body length (vs. shorter, ca. 14 mm), having a longer immovable finger or longer process of telopoditomere 2 (vs. shorter) and having a truncate/round central margin of subanal plate (vs. convex).

Based on material described by Likhitrakarn et al. 2021, deposited in the CUMZ (holotype CUMZ-Zeph0003, paratypes CUMZ-Zeph0004).

#### Zephronia viridisoma

Taxon classificationAnimaliaSphaerotheriidaZephroniidae

Rosenmejer & Wesener, 2021

68B73F83-9FCD-5EB1-B182-44C968D7F49B

Zephronia viridisoma Rosenmejer & Wesener, 2021 in Rosenmejer et al. 2021: 121.

##### Distribution and habitats.

The type locality is in Thailand (Nakhon Si Thammarat Province, Sichon District, Khao Lark Waterfall, 9°03'"N, 99°47'"E). Khao Lark Waterfall = Khao Lak = near Si Khit Waterfall. The material was collected from a dense jungle in limestone areas (Rosenmejer et al. 2021).

##### Remarks.

Only nine specimens have been collected and all were found to appear in a small area. This species can be regarded as endemic to southern Thailand.

#### Zephronia enghoffi sp. nov.

Taxon classificationAnimaliaSphaerotheriidaZephroniidae

C2FB91E9-1C25-5211-A435-2BAFDF617117

http://zoobank.org/033601FE-A945-445F-AE11-7CFEE3E05747

Figures 2A, B ; 7 ; 8 ; 13C, D ; 14B

##### Type material.

***Holotype***: Thailand – Khon Kaen Province • ♂; Tham Phaya Nakharat; 16°48'52"N, 101°57'16"E; 528 m a.s.l.; 21 July 2020; R. Srisonchai, C. Sutcharit leg.; CUMZ-Zeph0005. ***Paratypes***: Thailand – Khon Kaen Province • 4 ♂♂ 3 ♀♀; same locality as holotype; CUMZ-Zeph0006 • 2 ♀♀; same Province, Chum Pae District, Tham Poo Lup; 16°49'45.4"N, 101°59'7.6"E; 346 m a.s.l.; 10 October 2014; R. Srisonchai, C. Sutcharit leg.; CUMZ-Zeph0006. **Further specimens, not paratypes**: Thailand – Loei Province • 1 ♂ 2 ♀♀; Wang Saphung District, Pak Puan Arboretum; 17°21'20"N, 101°44'59"E; 316 m a.s.l.; 10 October 2014; R. Srisonchai, C. Sutcharit leg.; CUMZ-Zeph0006.

##### Etymology.

This species is named after Henrik Enghoff from Natural History Museum of Denmark, University of Copenhagen, Denmark, the Danish myriapodologist who initiated an important research step on millipede studies for Thailand.

##### Diagnosis.

A member of *Zephronia* s. s. in which the position of Tömösváry’s organ located next to the aberrant ommatidia, not inside the antennal groove. Adult body length medium, > 29 mm, usually ca. 32 mm, up to 36 mm; body brown or dark brown, inner surface (underside) of anal shield with a single locking carina on each side, and leg-pair 2 of male coxa with a long membranous lobe at mesal margin. Similar in these respects to *Z.golovatchi*sp. nov., but differs from this species by the following combination of characters; antenna short, leg-pair 2 of female coxa apico-mesally with large and conspicuous coxal ridge, operculum of vulva regularly rounded and narrow in posterior view, mesal margin of operculum tapering apically, central margin (tip) of subanal plate shallowly concave, process of telopoditomere 2 of anterior telopod quite long and equal in length to the combination of telopoditomeres 3+4, and immovable finger telopoditomere 2 of posterior telopod (process of telopoditomere 2) equal in length to movable finger (consisting of telopoditomeres 3 and 4).

##### Description.

***Body length***: Length in male 29.0–33.0 mm (holotype 31.0 mm), female 30.0–36.0 mm; head 4.5–5.5 mm; thoracic shield 5.5–7.0 mm; anal shield 11.0–11.5 mm.

***Body width***: Width in male 16.0–18.5 mm (holotype 18.0 mm), female 16.0–19.0 mm; head 9.0–10.0 mm; thoracic shield 16.0–18.0 mm; anal shield 14.0–17.0 mm.

***Body height***: Height in male 10.0–12.0 mm (holotype 11.0 mm), female 10.0–13.0 mm; thoracic shield 10.0–12.0 mm; tergite 10.0–13.0 mm.

***Color*** (Fig. 2A, B): Specimens in life with light brown to brown color; antennae dark brown; head, thoracic shield, tergites, paratergites and basal part of legs brown; posterior margin of tergites dark brown; a few apical podomeres greenish brown. Color in alcohol after 8 months not changed.

***Head***: Wide and stout, subtrapeziform; anterior part of head with dense and long setae; central part of head glabrous; posterior part of head with dense and short setae. Labrum with a single tooth at anterior margin. Each eye with 90–100 ommatidia. Aberrant ocellus located near antennal groove (at upper part of groove).

**Figure 3.**
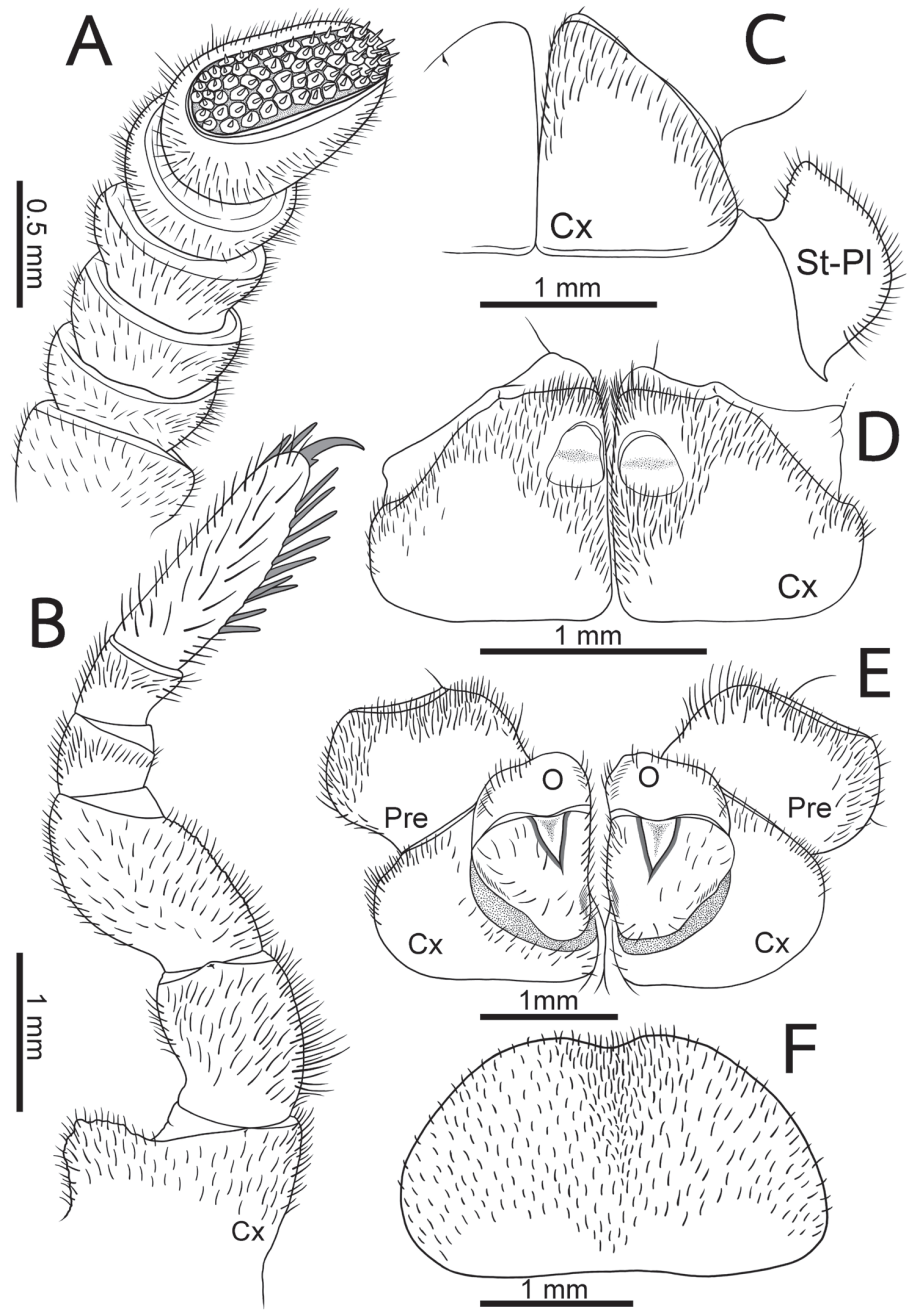
*Zephroniasiamensis***A–C** male **D, E** female (CUMZ-Zeph0013) **A** right antenna, ventral view **B** the ninth left leg, posterior view **C** first coxae with stigmatic plates, posterior view **D** coxae of second legs with gonopores, posterior view **E** coxae and prefemur of second legs with vulvae, posterior view **F** subanal plate, ventral view. Abbreviations: cx = coxa, o = operculum, pre = prefemur, syn-cx = syncoxite, St-Pl = stigmatic plate.

***Antenna*** (Fig. 7A): Short and stout, with rounded joints; length 3.5–4 mm; reaching backward to tarsus of legs 2 or 3. Lengths of antennomeres 6 > 5 > 4 = 3 = 2 = 1. Antennomere 6 densely setose, sensilla basiconica surrounding apical disc. Last antennomere thickened and flattened, strongly widened apically, axe-shaped. Shape of antennae sexually dimorphic; thickened, widened apically and slightly flattened in male, in female cylindrical. Apical disc with ca. 75 apical cones. No sclerotized ridge between antennal socket and ommatidia.

**Figure 4.**
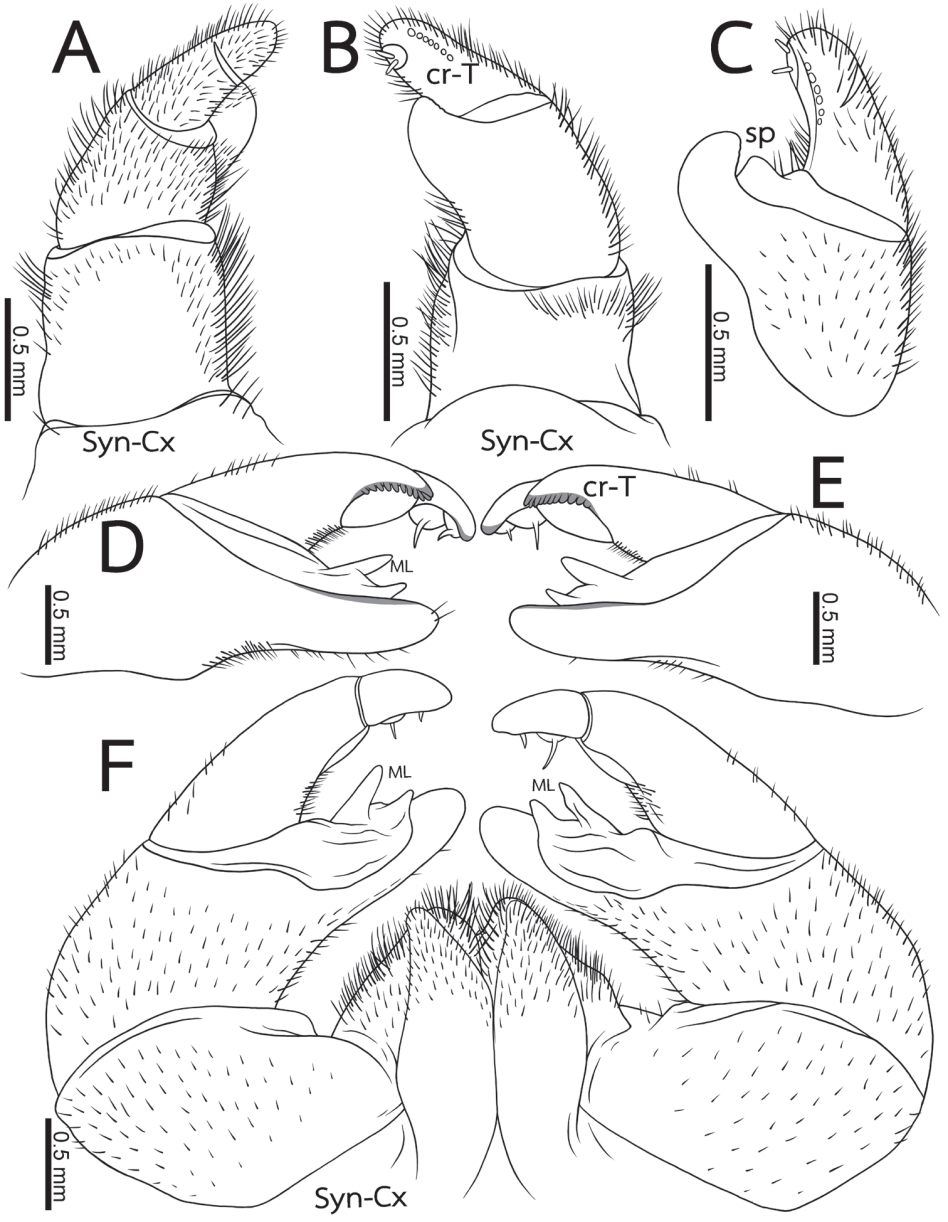
*Zephroniasiamensis***A–C** left anterior telopod, anterior, posterior and sublateral views, respectively **D, E** Left and right posterior telopods, posterior view **F** Posterior telopod, anterior view. Abbreviations: cr-T = crenulated teeth, cx = coxa, ML = membranous lobe, sp = sclerotized process, syn-cx = syncoxite.

***Tömösváry’s organ***: Separated from ommatidium, located on a brim between ommatidia and antennal socket, smaller in diameter than an individual ommatidium.

***Gnathochilarium***: Ventral surface with setae, other structures typical of the order. Mandibles not dissected.

**Figure 5.**
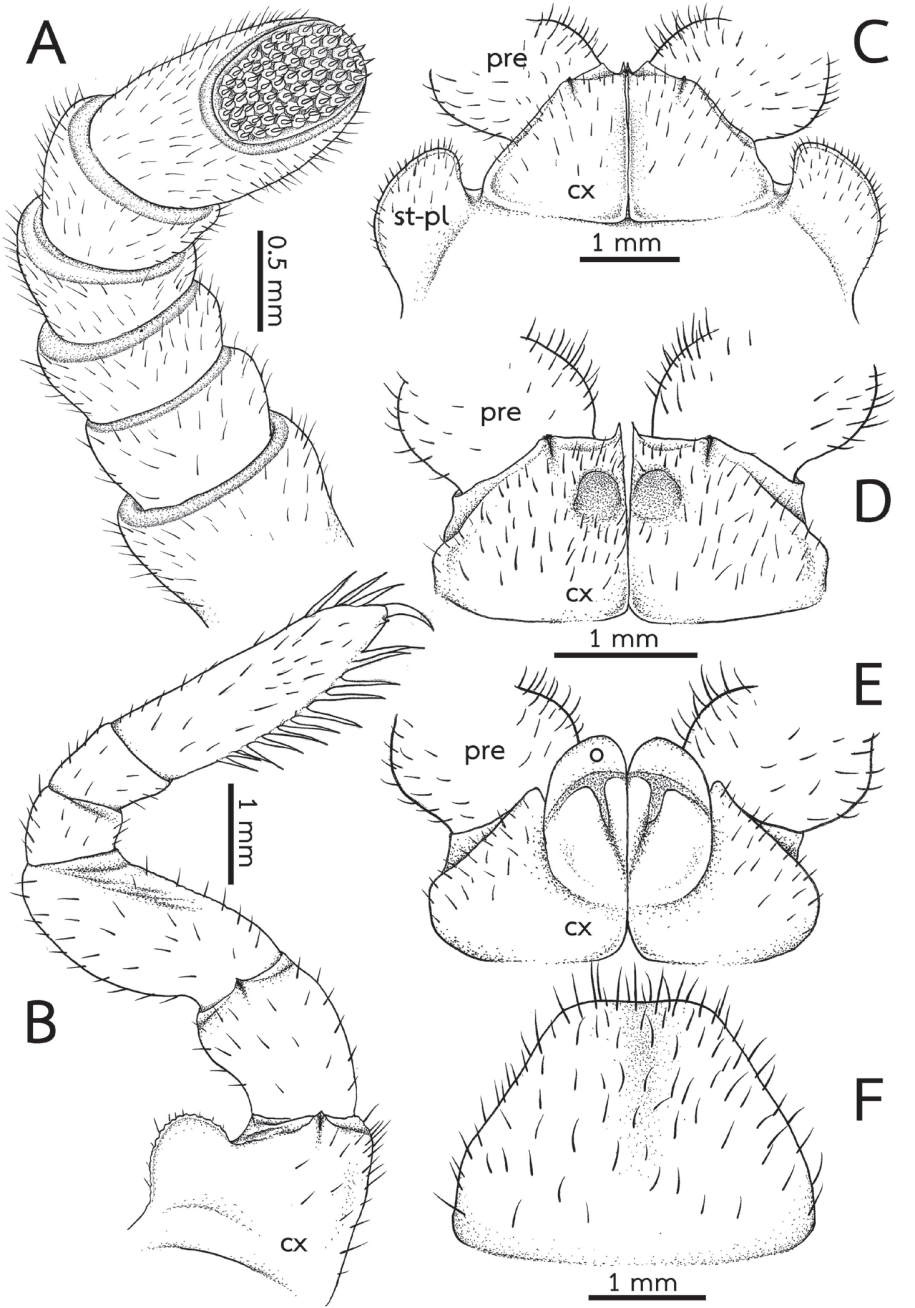
*Zephroniaphrain***A–D** male specimen from Phawor Shrine, Tak Province **E, F** female **A** right antenna, ventral view **B** the ninth left leg, posterior view **C** first coxae with stigmatic plates, posterior view **D** coxae of second legs with gonopores, posterior view **E** coxae and prefemur of second legs with vulvae, posterior view **F** subanal plate, ventral view. Abbreviations: cx = coxa, o = operculum, pre = prefemur, syn-cx = syncoxite, st-pl = stigmatic plate.

***Stigmatic plates*** (Fig. 7C): First stigmatic plate subtriangular; apex rounded, broad, expanded apically then becoming a fanlike; curved towards coxa 1.

**Figure 6.**
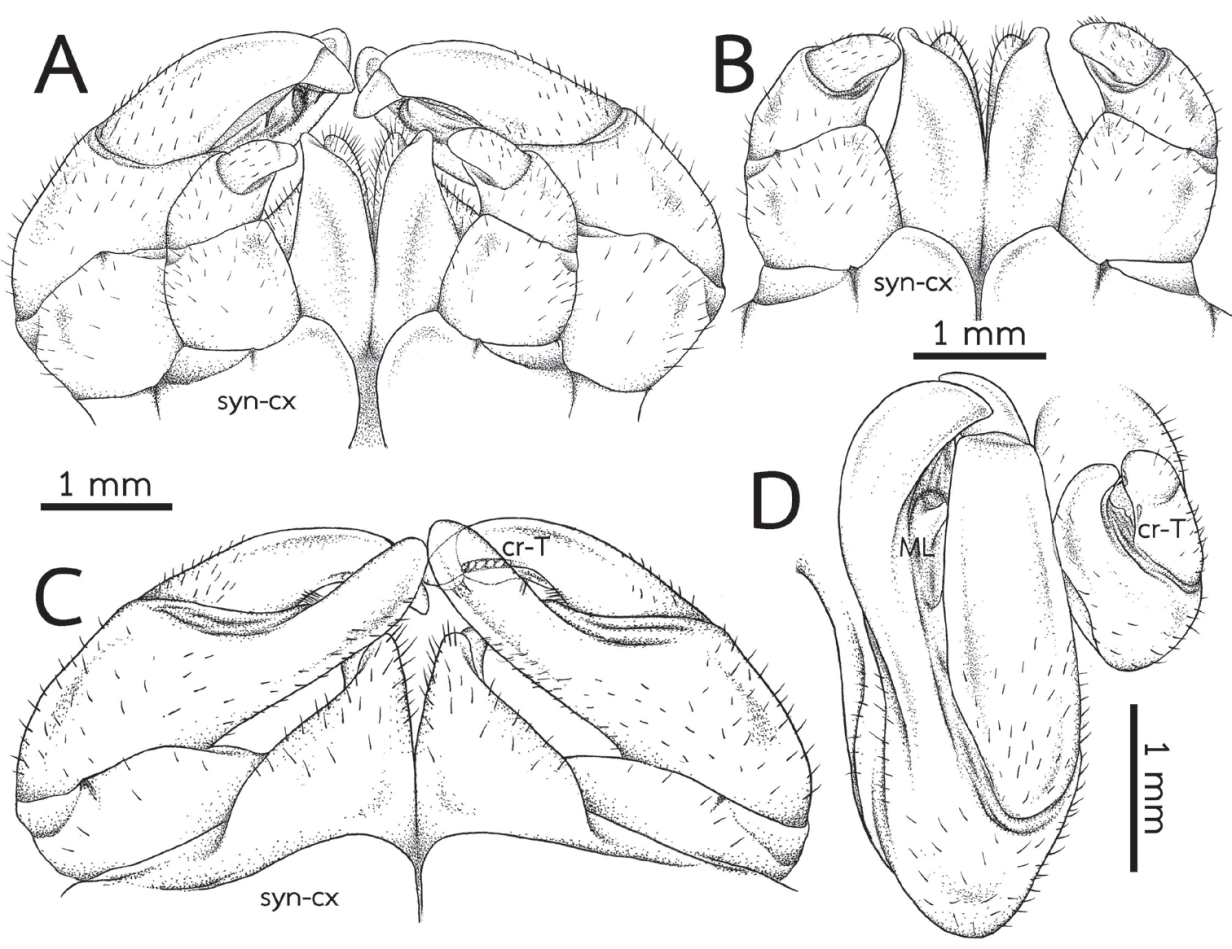
*Zephroniaphrain*, male specimen from Phawor Shrine, Tak Province **A** telopods, anterior view **B** anterior telopod, anterior view **C** posterior telopod, posterior view **D** right anterior and posterior telopods, ventral view. Abbreviations: cr-T = crenulated teeth, cx = coxa, ML = membranous lobe, syn-cx = syncoxite.

***Laterotergites***: Laterotergite 1 narrow, projecting into a sharp tip. Laterotergite 2 larger than laterotergite 1, tip weakly extended, with a round projection.

***Collum***: Surface glabrous, except for anterior margins near rim with isolated and long setae.

***Thoracic shield***: Surface as those of tergites, covered with small setae, each seta located in a tiny pit; shallow groove wide anterolaterally, with very long setae.

***Tergites*** (Fig. 2A, B): Quite shiny; surface densely setose, visible in normal vision; entirely covered by short setae, each locating in tiny pits; tip of paratergite of midbody tergites curved, directed posteroventrad.

***Endotergum*** (Figs 13C, D, 14B): Posterior margin flat, regular. Inner section (inner area) with setiferous tubercles or setae. Middle section (middle area) with a single row of elliptical cuticular impressions, distance between impressions longer than individual diameter. Bristles arranged in two rows, tip of the longest bristles extended beyond posterior margin or reaching to posterior margin.

***Anal shield***: Sexually dimorphic, in female very large and strongly rounded, in male slightly more rectangular. Outer surface covered by tiny and dense setae locating in small pits, similar to those of tergites. Inner surface (underside) covered by long setae; with a single, black, and long locking carina, half as long as length of last laterotergite.

**Figure 7.**
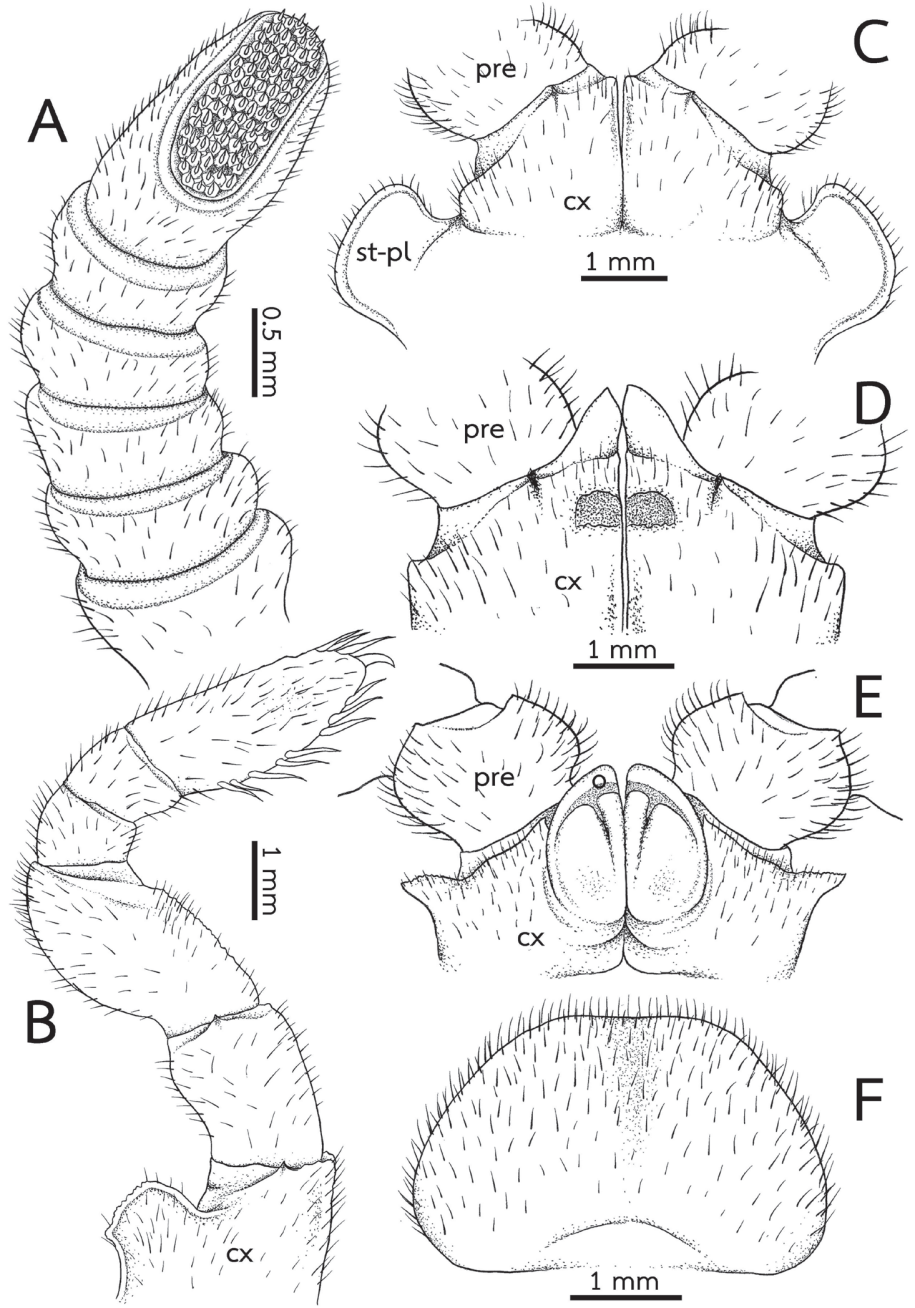
*Zephroniaenghoffi*sp. nov. **A–D** male holotype (CUMZ-Zeph0005) **E, F** female paratypes (CUMZ-Zeph0006) **A** right antenna, ventral view **B** the ninth left leg, posterior view **C** First coxae with stigmatic plates, posterior view **D** coxae of second legs with gonopores, posterior view **E** coxae and prefemur of second legs with vulvae, posterior view **F** subanal plate, ventral view. Abbreviations: cx = coxa, o = operculum, pre = prefemur, syn-cx = syncoxite, st-pl = stigmatic plate.

***Legs*** (Fig. 7B): Leg-pairs 1 and 2 without apical spine. Leg-pairs 1 with 2 ventral spines, leg-pair 2 with four ventral spines. Leg-pair 3 with 5–7 ventral spines and one apical spine. Leg-pair 4 with 8–11 ventral spines, and one or two apical spines. Leg-pairs 5–19 with 8–10 ventral spines and 1–3 apical spines. Last two leg-pairs with 7–10 ventral spines and one or two apical spines. In leg 9, femur ca. 1.5×, tarsus ca. 2.3× longer than wide. Length of tarsus ≥ femur > prefemur > coxa > tibia ≥ postfemur. All podomeres densely setose. Coxa large, with dentate ridge marginally (coxal process). Coxal process absent in leg-pairs 1 and 2 (except for female leg-pair 2). Leg-pair 2 of female coxa apico-mesally with large, conspicuous coxal ridge, directed laterad. Leg-pair 2 of male coxa with membranous lobe at mesal margin; lobe large and long, projecting ventrad. Prefemur without teeth. Femur rather short and stout, slightly extended mesally, mesal margin with 5–7 small teeth.

***Subanal plate*** (Fig. 7F): Subsemicircular, undivided, wide; central margin (tip) shallowly concave, broad; lateral margin slightly convex. Densely setose.

***Male sexual characters*** (Fig. 7D): Gonopore quite large, covered with a single, undivided, subsemicircular, sclerotized plate.

**Figure 8.**
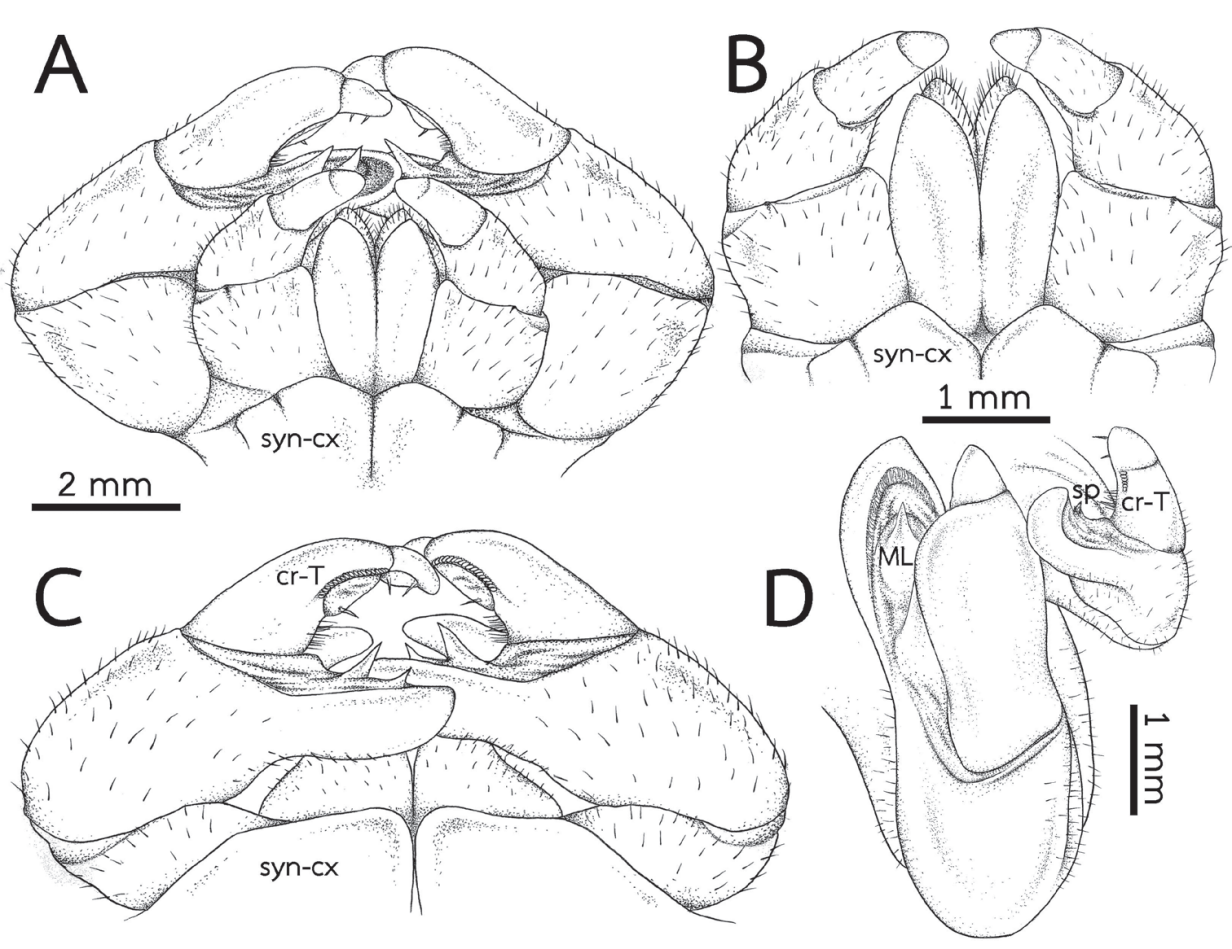
*Zephroniaenghoffi*sp. nov., male holotype (CUMZ-Zeph0005) **A** telopods, anterior view **B** anterior telopod, anterior view **C** posterior telopod, posterior view **D** right anterior and posterior telopods, ventral view. Abbreviations: cr-T = crenuations/teeth, cx = coxa, ML = membranous lobe, sp = sclerotized process, syn-cx = syncoxite.

***Anterior telopods*** (Fig. 8A, B, D): Telopodite with four telopoditomeres; all telopoditomeres sparsely setose, except for the apical part of telopoditomere 3 and all parts of telopoditomere 4 without setae. First telopoditomere rectangular, slightly large and stout, broader than telopoditomeres 2–4. Telopoditomere 2 large. Process of telopoditomere 2 equal in length to the combination of telopoditomeres 3 and 4; visible in posterior view; curved and slender, 1.5X longer than wide, twice as long as telopoditomere 4; tip bent and round, directed anteriad, close to the basal part of telopoditomere 4. Margin towards telopoditomere 3 with a membranous area carrying a sclerotized process (sp), conspicuous, short, apically with sharp tip. Telopoditomere 3 with six small crenulated teeth (cr-T) in ventral side. Telopoditomere 4 short and stout, conspicuous, straight; tip round, directed mesad; with two prominent sclerotized spines in posterior side.

***Posterior telopods*** (Fig. 8A, C, D): Telopodite with four telopoditomeres; telopoditomeres 1 and 2 on both sides covered with sparse setae, except for immovable finger part; telopoditomere 3 at base of both inner margin and outer margin with a few setae; telopoditomere 4 without setae. First telopoditomere stout and broad, half as long as telopoditomere 2. Telopoditomere 2 slender, immovable finger (process of telopoditomere 2) as long as movable finger (consisting of telopoditomeres 3 and 4). Immovable finger long and slender, wide, 2.5× as long as wide, not curved, tip directed mesad; at margin with several semi-circular rows of sclerotized spots. Margin towards movable finger with two conspicuous membranous lobes, triangular, inner lobe bigger and longer than outer one. Telopoditomere 3 slender, quite long, slightly expanded distad, slightly curved, thrice as long as telopoditomere 4; with a long and sclerotized spine located on a large, swollen, membranous lobe; posterior part with a row of 17–19 crenulated teeth (cr-T). Telopoditomere 4 short and stout, 1.5× longer than wide; at inner margin with a large, conspicuous, swollen, membranous lobe and two evident sclerotized spines.

***Female sexual characters*** (Fig. 7E): Vulva large, covering ca. 2/3 coxa, located at mesal side, extending mesally to basal third of prefemur. Operculum regularly rounded, narrow in posterior view; mesal margin not protruding.

##### Distribution and habitats

(Figs 15B, C, 16). All specimens were collected from limestone habitats (in dry dipterocarp forest). Known only from three sites in limestone mountain ranges of Khon Kaen and Loei provinces.

**Remarks.** With regard to the morphological characters of coxae 2 in both male and female, this species exhibits the remarkable shape in which the male has a very long membranous lobe (Fig. 7D) and the female displays conspicuous coxal ridges apico-mesally (Fig. 7E). Moreover, the surface of tergites covered with conspicuous setae/hairy in *Z.enghoffi*sp. nov. is more distinctive than in the other two new species (Fig. 2A, B).

#### Zephronia golovatchi sp. nov.

Taxon classificationAnimaliaSphaerotheriidaZephroniidae

BFF35D3E-5452-5997-A0DD-59DD291AE0F0

http://zoobank.org/8033D6ED-BAE9-4347-851D-8B2C7B147FE5

**Figure 9.**
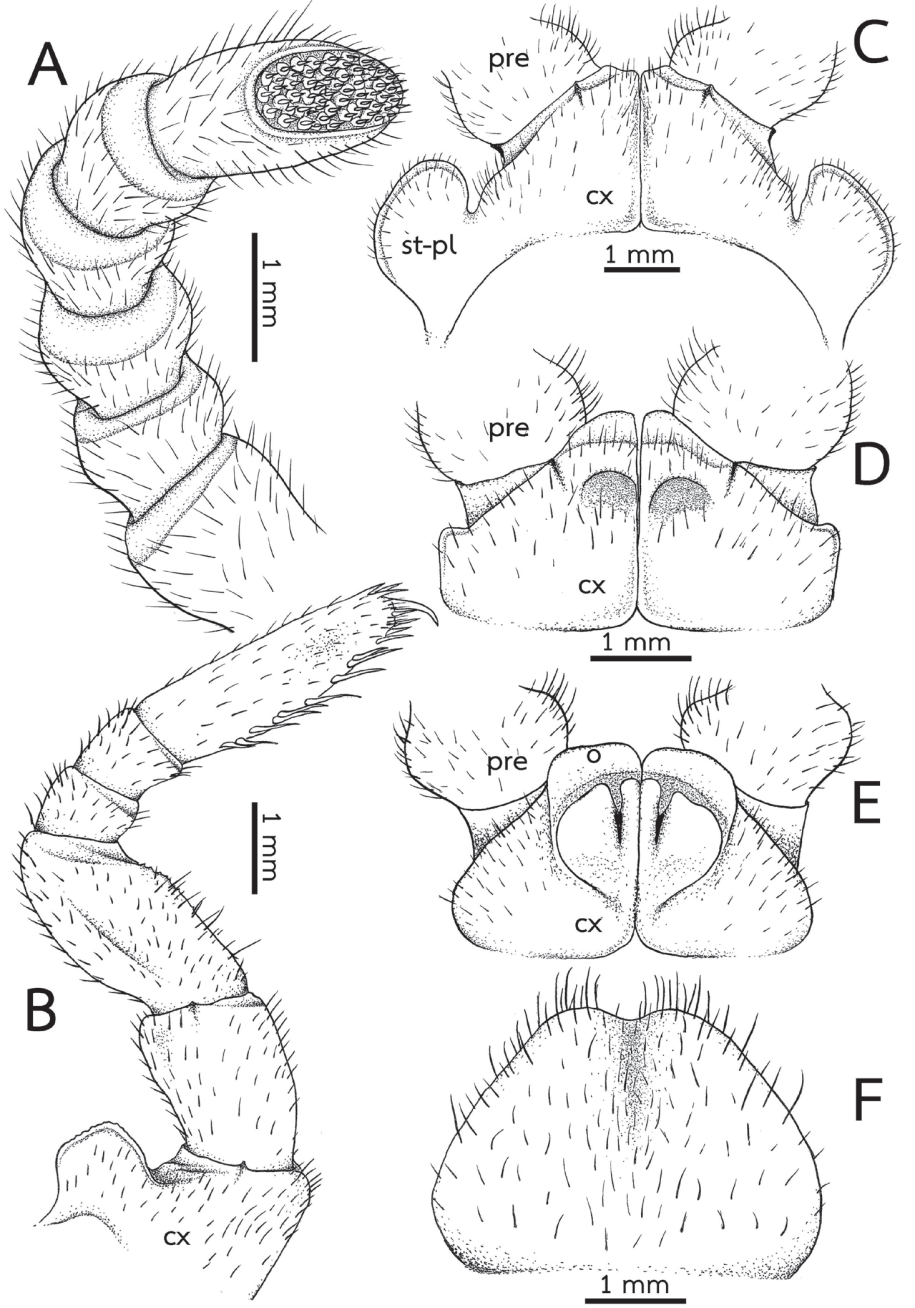
*Zephroniagolovatchi*sp. nov. **A–D** male holotype (CUMZ-Zeph0007) **E, F** female paratypes (CUMZ-Zeph0008) **A** right antenna, ventral view **B** the ninth left leg, posterior view **C** first coxae with stigmatic plates, posterior view **D** coxae of second legs with gonopores, posterior view **E** coxae and prefemur of second legs with vulvae, posterior view **F** subanal plate, ventral view. Abbreviations: cx = coxa, o = operculum, pre = prefemur, syn-cx = syncoxite, st-pl = stigmatic plate.

**Figure 10.**
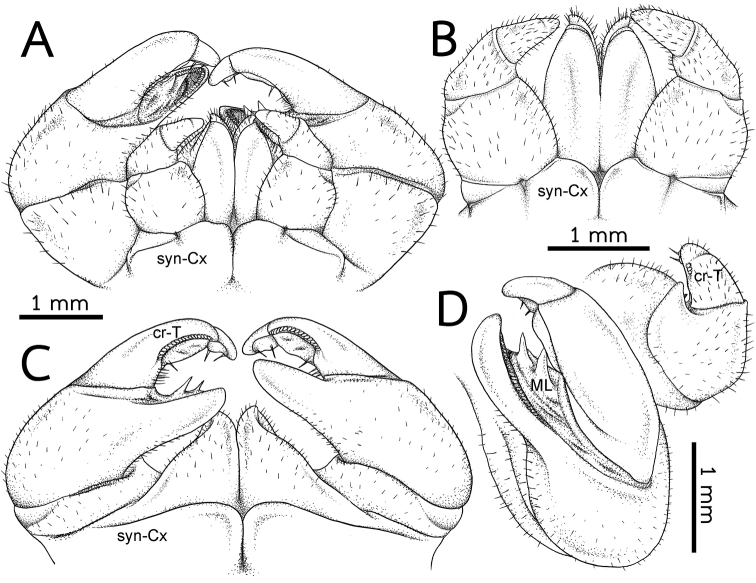
*Zephroniagolovatchi*sp. nov., male holotype (CUMZ-Zeph0007) **A** telopods, anterior view **B** anterior telopod, anterior view **C** posterior telopod, posterior view **D** right anterior and posterior telopods, ventral view. Abbreviations: cr-T = crenulated teeth, cx = coxa, ML = membranous lobe, syn-cx = syncoxite.

Figures 2C, D ; 9 ; 10 ; 13E, F ; 14C

##### Type material.

***Holotype***: Thailand – Nakhon Ratchasima Province • ♂; Pak Chong District, Khao Yai National Park, Khao Luk Chang; 14°31'49.6"N, 101°21'32"E; 410 m a.s.l.; 26 April 2009; N. Likhitrakarn, C. Sutcharit, W. Siriwut leg.; CUMZ-Zeph0007. ***Paratypes***: Thailand – Nakhon Ratchasima Province • 1 ♂ 4 ♀♀; same locality as holotype; CUMZ-Zeph0008.

##### Etymology.

The species is named for our highly esteemed colleague Sergei I. Golovatch (Zoological Museum, State University of Moscow, Russia), one of the most productive millipede taxonomists, who encouraged all new and young myriapodologists in Thailand.

##### Diagnosis.

Adult body length medium to large > 29 mm, usually 35 mm, up to 37 mm; body brown or dark brown, marginal bristles of endotergum extending over posterior margin, inner surface (underside) of anal shield with a single locking carina on each side, and leg-pair 2 of male coxa with membranous lobe at mesal margin. Similar in these respects to *Z.enghoffi*sp. nov., but differs from this species by the following combination of characters: antenna long; operculum of vulva regularly rounded and broad in posterior view; mesal margin of operculum not tapering apically; central margin (tip) of subanal plate divided by a conspicuous mesal constriction, process of telopoditomere 2 of anterior telopods shorter than telopoditomere 3; telopoditomere 3 of anterior telopods with 2 or 3 crenulated teeth; immovable finger telopoditomere 2 of posterior telopod (process of telopoditomere 2) shorter than movable finger (consisting of telopoditomeres 3 and 4).

##### Description.

***Body length***: Length in male 35.0–36.5 mm (holotype 35.0 mm), female 35.0–37.0 mm; head 5.5–7.5 mm; thoracic shield 5.0–6.0 mm; anal shield 10.5–11.5 mm.

***Body width***: Width in male 19.0–21.0 mm (holotype 20.0 mm), female 19.0–22.0 mm; head 10.0–11.0 mm; thoracic shield 17.5–20.5 mm; anal shield 16.0–18.5 mm.

***Body height***: Height in male ca 11.0 mm (holotype 11.0 mm), female 11.0–12.0 mm; thoracic shield 9.0–10.5 mm; tergite 10.0–11.5 mm.

***Color*** (Fig. 2C, D): Specimens in life with brown color; antennae dark brown; head, collum, thoracic shield, tergites, paratergites, anal shield and legs brown; posterior margin of tergites dark brown. Color in alcohol after 13 years changed to pale brown.

***Head***: Wide and stout, subtrapeziform; anterior part of head with dense and long setae; central part of head with sparse and long setae; posterior part of head with dense and short setae. Labrum with a single tooth at anterior margin. Each eye with ca. 90–100 ommatidia. Aberrant ocellus located inside antennal groove (at upper part of groove).

***Antenna*** (Fig. 9A): Quite long and stout, with rounded joints; length ca. 5 mm; reaching backward to tarsus of legs 3 or 4. Lengths of antennomeres 6 > 5 = 4 = 3 = 2 = 1. Antennomere 6 densely setose, sensilla basiconica surrounding apical disc. Last antennomere thickened and flattened, strongly widened apically, axe-shaped. Shape of antennae sexually dimorphic; thickened, widened apically and slightly flattened in male, in female cylindrical. Apical disc with 90–100 apical cones. No sclerotized ridge between antennal socket and ommatidia.

***Tömösváry’s organ***: Not distinctly separated from ommatidium, located closely to anterior margin of ommatidia, equal in size to an individual ommatidium.

***Gnathochilarium***: Ventral surface with setae, other structures typical of the order. Mandibles not dissected.

***Stigmatic plates*** (Fig. 9C): First stigmatic plate subtriangular; apex rounded, broad; slightly projecting towards coxa 1.

***Laterotergites***: Laterotergite 1 narrow, projecting into a sharp tip. Laterotergite 2 broader than laterotergite 1, tip slightly extended, with round projection.

***Collum***: Surface glabrous, except for anterior margins near rim with isolated and long setae.

***Thoracic shield***: Surface as those of tergites, covered with inconspicuous and small setae, each seta located in tiny pits; shallow groove wide anterolaterally, with very long setae.

***Tergites*** (Fig. 2C, D): Quite dull; surface entirely covered by short setae, visible by normal vision; each seta locating in tiny pits; anterior margin densely setose; posterior margin sparsely setose; tip of paratergite in midbody slightly curved, directed posteroventrad.

***Endotergum*** (Figs 13E, F, 14C): Posterior margin flat, regular. Inner section (inner area) with a few setiferous tubercles or setae. Middle section (middle area) with a single row of small, conspicuous, elliptical cuticular impressions; distance between impressions longer than individual diameter; with a row of conspicuous ridges between bristles and impressions. Bristles arranged in two rows, tip of the longest bristles extended beyond posterior margin or reaching to posterior margin.

***Anal shield***: Sexually dimorphic, in female very large and weakly bell-shaped, in male slightly bell-shaped. Outer surface pubescent, similar to those of tergites; setae small and very short locating in tiny pits; anterior margin densely setose, posterior margin sparsely setose. Inner surface (underside) covered by setae; with a single, black, very long, locking carina, ca. 1/3 as long as length of last laterotergite.

***Legs*** (Fig. 9B): Leg-pairs 1 and 2 without apical spine. Leg-pair 1 with two ventral spines, leg-pair 2 with four or five ventral spines. Leg-pair 3 with 7–9 ventral spines and one or two apical spines. Leg-pair 4 with 9–11 ventral spines and one or two apical spines. Leg-pairs 5–19 with 8–12 ventral spines and 1–3 apical spines. Last two leg-pairs with 9–11 ventral spines and one or two apical spines. In leg 9, femur ca. 1.7×, tarsus ca. 3.2× longer than wide. Length of tarsus > femur > prefemur > coxa > tibia ≥ postfemur. All podomeres densely setose. Coxa large, with dentate ridge marginally (coxal process). Coxal process absent in leg-pairs 1 and 2. Prefemur without teeth. Femur extended mesally, mesal margin with 7–9 conspicuous teeth.

***Subanal plate*** (Fig. 9F): Trapeziform, divided by a conspicuous mesal constriction; central margin (tip) strongly concave, narrow; lateral margin straight. Densely setose.

***Male sexual characters*** (Fig. 9D): Gonopore large, covered with a single, undivided, subsemicircular, sclerotized plate.

***Anterior telopods*** (Fig. 10A, B, D): Telopodite with four telopoditomeres; telopoditomeres 3 and 4 often clearly divided by conspicuous suture, some specimens inconspicuous; all telopoditomeres sparsely setose, except for process of telopoditomere 2 with no setae. First telopoditomere rectangular, large, stout. Telopoditomere 2 slender. Process of telopoditomere 2 short, shorter than telopoditomeres 3; visible in posterior view; tip curved, blunt and narrow, directed anteromesad, close to middle part of telopoditomere 3. Margin towards telopoditomere 3 with a membranous area carrying a sclerotized process (sp); a process inconspicuous, short, tip quite sharp. Telopoditomere 3 with two or three crenulated teeth (cr-T), conspicuous. Telopoditomere 4 very short and stout, inconspicuous; tip round, directed mesad; with two small, conspicuous, sclerotized spines in posterior side.

***Posterior telopods*** (Fig. 10A, C, D): Telopodite with four telopoditomeres; telopoditomeres 1 and 2 on both sides sparse setose, except for apical part of immovable finger (process of telopoditomere 2); telopoditomere 3 at base of inner margin with conspicuous setae, but none for outer margin; telopoditomere 4 without setae. First telopoditomere large, wide, as long as wide. Telopoditomere 2 large, immovable finger (process of telopoditomere 2) relatively shorter than movable finger (consisting of telopoditomeres 3 and 4). Immovable finger slender, twice as long as wide, slightly curved, tip directed anteroventrad; at margin with several semi-circular rows of sclerotized spots, conspicuous. Margin towards movable finger with two membranous lobes, conspicuous and long, conical, inner lobe bigger and longer than outer one, tip sharp. Telopoditomere 3 very long and slender, tapering apically, strongly curved, thrice as long as telopoditomere 4; with a long and sclerotized spine located on a large, swollen, membranous lobe; posterior part with a row of 17 or 18 crenulated teeth (cr-T). Telopoditomere 4 slender, 2× longer than wide; at inner margin with a large, conspicuous, swollen, membranous lobe and with two evident sclerotized spines; tip curving mesodorsad.

***Female sexual characters*** (Fig. 9E): Vulva large, covering ca. 2/3 coxa, located at mesal side, extending mesally to basal third of prefemur. Operculum regularly rounded, margin straight, mesal margin not protruding.

##### Distribution and habitats

(Fig. 16). Known only from the type locality. All specimens have been taken from limestone habitats and were found walking on top of decayed wood or hiding under leaf litter.

##### Remarks.

This species has thin membranous lobe on male coxae 2 (Fig. 9D), but this lobe seems to be shorter than that of *Z.enghoffi*sp. nov. (Fig. 7D).

#### Zephronia panhai sp. nov.

Taxon classificationAnimaliaSphaerotheriidaZephroniidae

4F74A9B6-32BE-573B-9E8F-7C21AAF9F879

http://zoobank.org/127730AA-2FEC-49F1-B3F9-412C216E7F53

**Figure 11.**
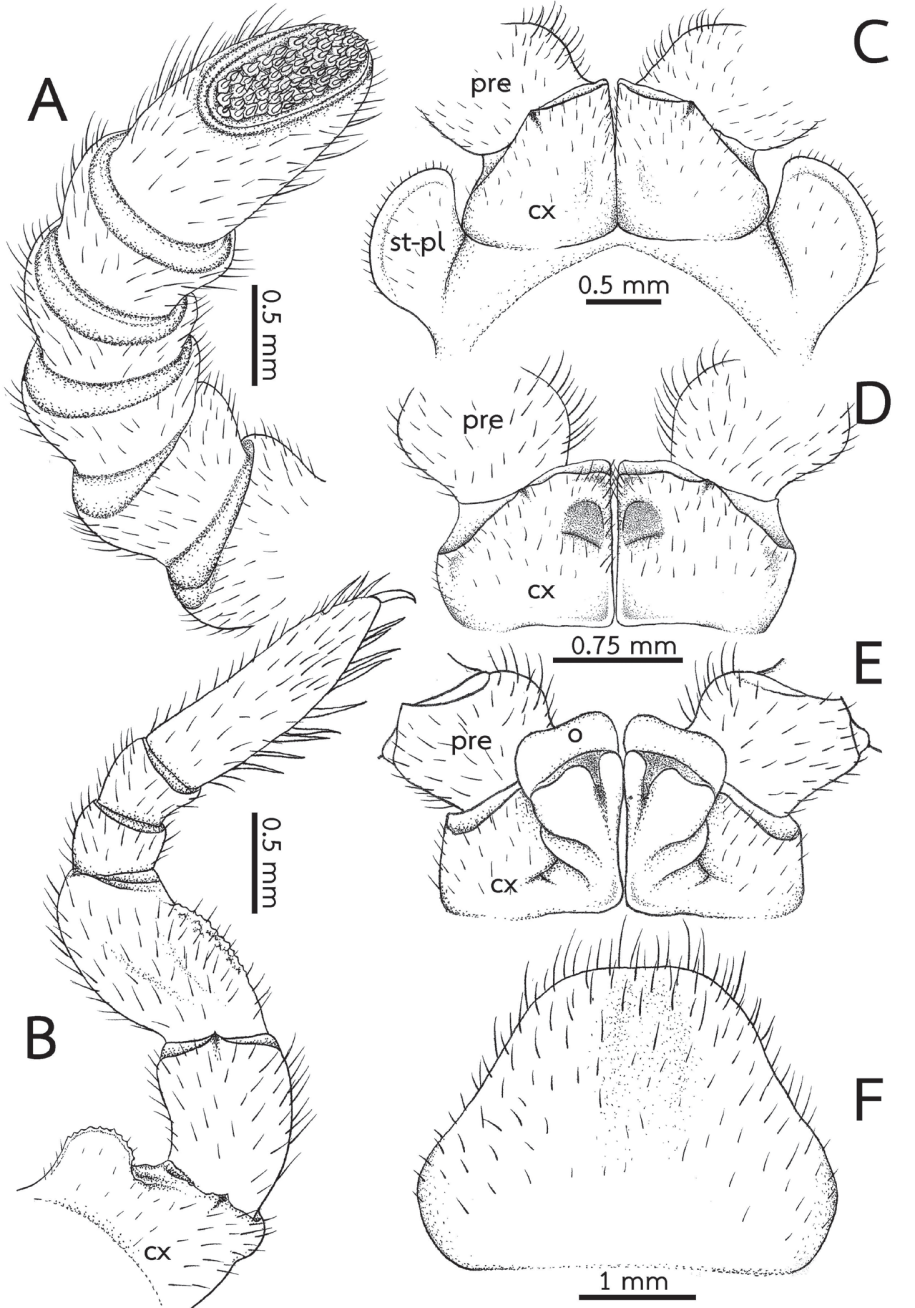
*Zephroniapanhai*sp. nov. **A–D** male holotype (CUMZ-Zeph0009) **E, F** female paratypes (CUMZ-Zeph0010) **A** right antenna, ventral view **B** the ninth left leg, posterior view **C** first coxae with stigmatic plates, posterior view **D** coxae of second legs with gonopores, posterior view **E** coxae and prefemur of second legs with vulvae, posterior view **F** subanal plate, ventral view. Abbreviations: cx = coxa, o = operculum, pre = prefemur, syn-cx = syncoxite, st-pl = stigmatic plate.

**Figure 12:**
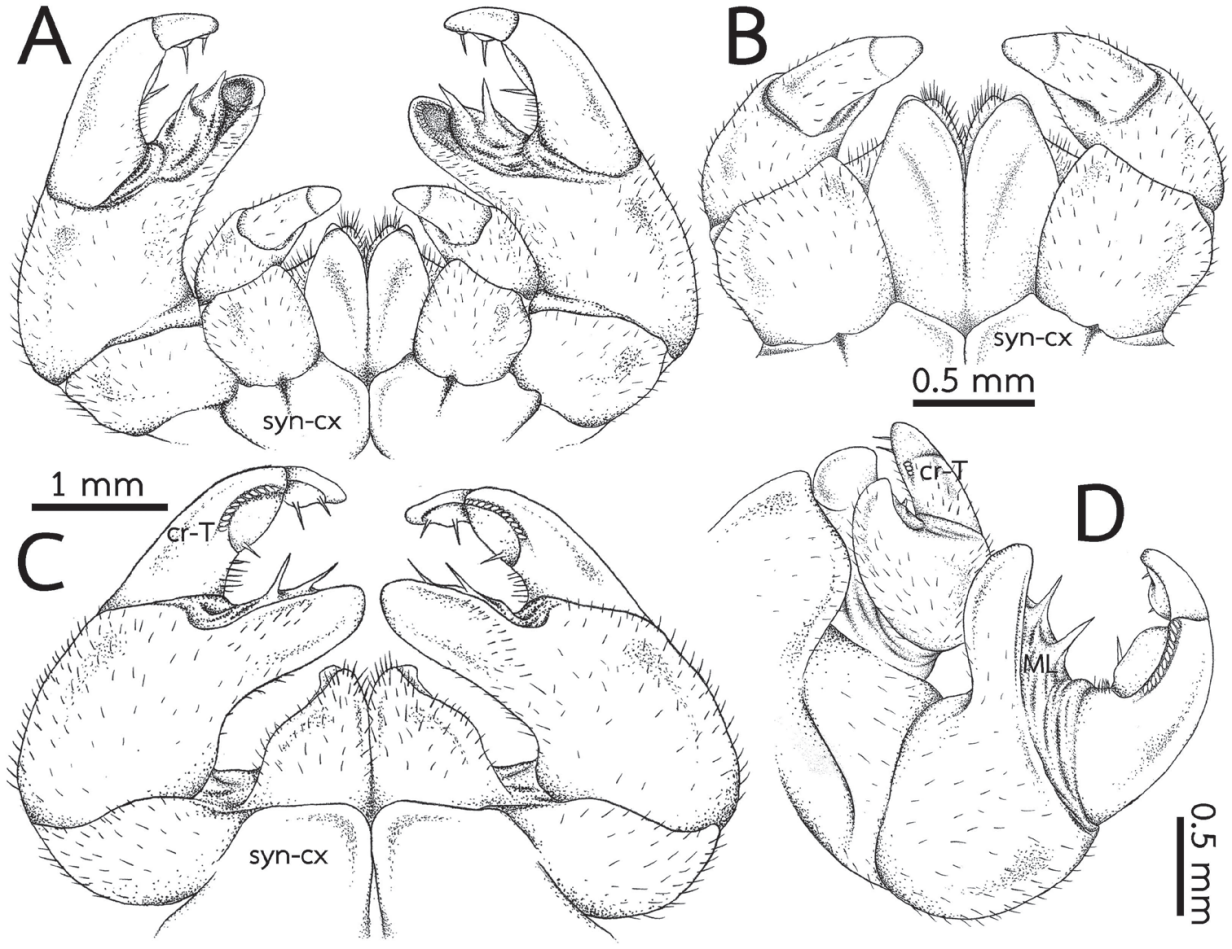
. *Zephroniapanhai*sp. nov., male holotype (CUMZ-Zeph0009) **A** telopods, anterior view **B** anterior telopod, anterior view **C** posterior telopod, posterior view **D** right anterior and posterior telopods, ventral view. Abbreviations: cr-T = crenulated teeth, cx = coxa, ML = membranous lobe, sp = sclerotized process, syn-cx = syncoxite.

**Figure 13.**
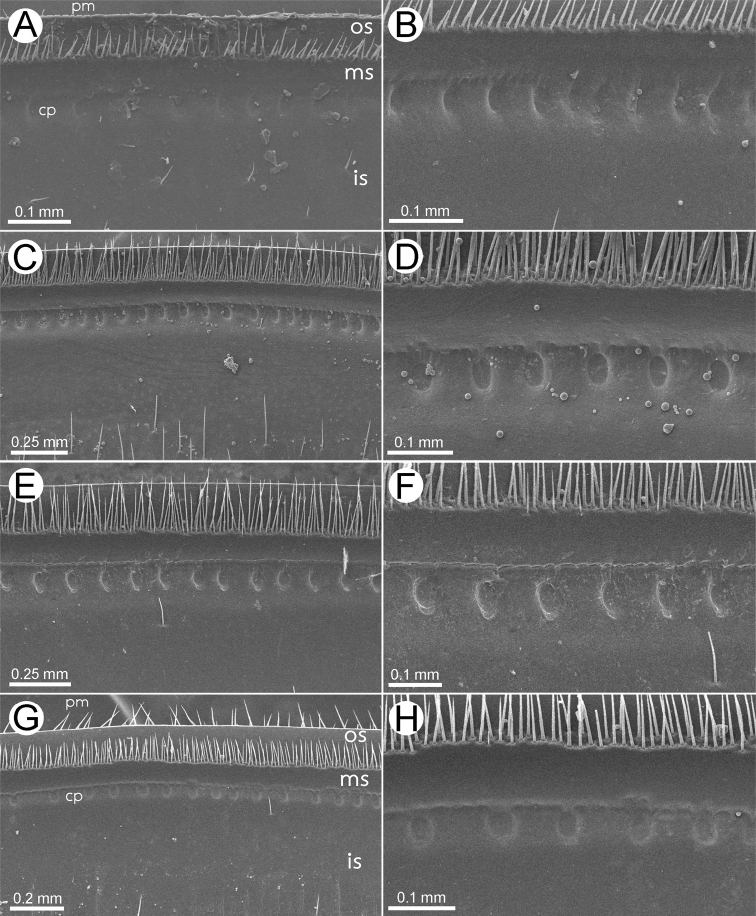
SEM of endoterga of body ring 7, all from ventral views **A, B***Zephroniasiamensis* Hirst, 1907 **C, D***Zephroniaenghoffi*sp. nov. **E, F***Zephroniagolovatchi*sp. nov. **G, H***Zephroniapanhai*sp. nov. Abbreviations: cp = cuticular impression, ms = middle section, is = inner section, os = outer section, pm = posterior margin.

**Figure 14.**
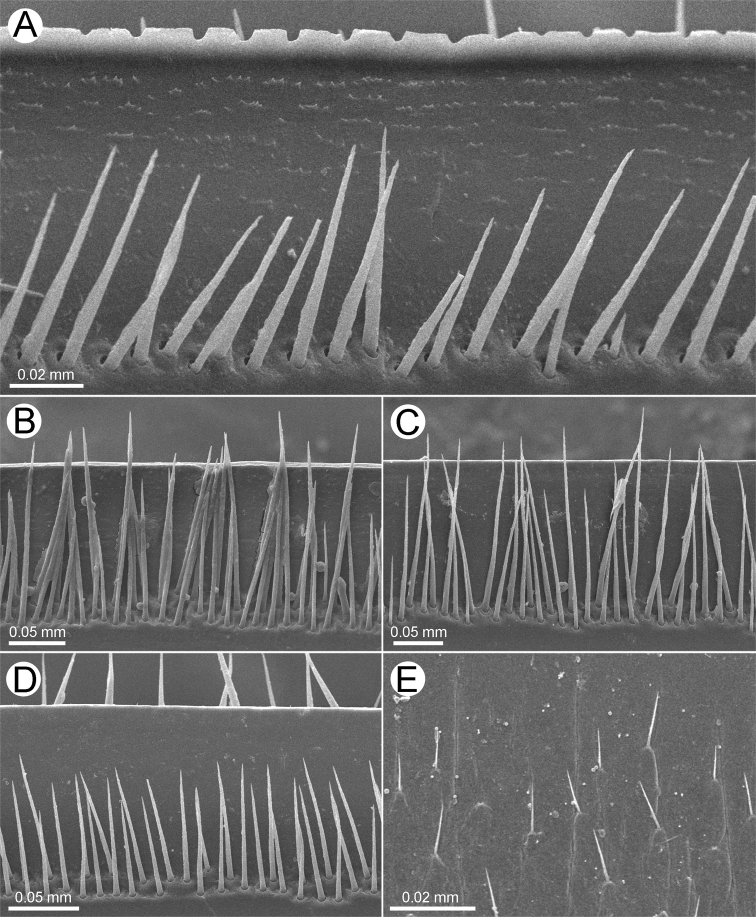
SEM of endoterga in body ring 7, all from ventral views **A***Zephroniasiamensis* Hirst, 1907 **B***Zephroniaenghoffi*sp. nov. **C***Zephroniagolovatchi*sp. nov. **D***Zephroniapanhai*sp. nov. **E** inner area of endotergum in *Zephroniapanhai*sp. nov.

Figures 2E, F ; 11 ; 12 ; 13G, H ; 14D, E

##### Type material.

***Holotype***: Thailand • ♂; Phetchaburi Province, Tha Yang District, Wat Khao Khachiu; 12°57'42.7"N, 99°54'49.9"E; 22 m a.s.l.; 17 August 2019; R. Srisonchai, C. Sutcharit, W. Siriwut leg.; CUMZ-Zeph0009. ***Paratypes***: Thailand – Phetchaburi Province • 8 ♂♂ 6 ♀♀; same locality as holotype; CUMZ-Zeph0010 • 1 ♂; same data as holotype; NHMD • 1 ♂; same data as holotype; ZMUM • 1 ♂; same data as holotype; ZRC. **Further specimens, not paratypes**: Thailand – Phetchaburi Province • 3 ♂♂ 2 ♀♀; Khao Yoi District, Wat Puangmali (Wat Tham Khao Ego); 13°18'45.3"N, 99°47'5.1"E; 22 m a.s.l.; 8 September 2016; R. Srisonchai, C. Sutcharit, W. Siriwut leg.; CUMZ-Zeph0010 • 1 ♂ 2 ♀♀; Rachaburi Province, Pak Tho District, Wat Buri Ratchawanaram; 13°22'45"N, 99°47'6"E, 26 m a.s.l.; 14 November 2019; R. Srisonchai, C. Sutcharit, W. Siriwut leg.; CUMZ-Zeph0010 • 2 ♂♂ 4 ♀♀; Kanchaburi Province, Mueang District, Wat Tham Mangkorn Thong; 13°59'8.2"N, 99°31'2.9"E; 46 m a.s.l.; 3 September 2017; R. Srisonchai, C. Sutcharit, W. Siriwut leg.; CUMZ-Zeph0010.

##### Etymology.

The species name recognizes the great professor and a long-time mentor to the authors, Somsak Panha (Chulalongkorn University Museum of Zoology, Thailand).

##### Diagnosis.

Differs from all congeners by the combination of the following characters; grey body color, adult body length ca. 21 mm, tergites covered by conspicuous setae, long setae on tergites extending over the posterior margin (Figs 13G, 14D), marginal bristles of endotergum not extending over posterior margin, margin of operculum on vulva slightly concave and slightly invaginated medially, telopoditomere 3 of anterior telopods with conspicuous crenulated teeth and telopoditomere 3 of posterior telopods with a row of 11or 12 crenulated teeth.

##### Description.

***Body length***: Length in male 19.0–22.0 mm (holotype 20.0 mm), female 20.0–23.0 mm; head 4.0 mm; thoracic shield 4.0–4.5 mm; anal shield 6.0–7.5 mm.

***Body width***: Width in male 10.0–11.5 mm (holotype 10.0 mm), female 10.0–12.0 mm; head 6.0–7.0 mm; thoracic shield 10.0–11.0 mm; anal shield 9.5–10.5 mm.

***Body height***: Height in male 7.0–7.5 mm (holotype 7.0 mm), female 7.0–7.5 mm; thoracic shield 6.0–7.0 mm; tergite 6.5–7.5 mm.

***Color*** (Fig. 2E, F): Specimens in life with light grey; head, antennae and collum greenish grey; thoracic shield, tergites and anterior part of anal shield grey; paratergites, posterior margins of tergites and posterior part of anal shield greyish brown. Color in alcohol after two years not changed.

***Head***: Wide and stout, subtrapeziform; anterior part of head with dense and long setae; central part of head with sparse and long setae; posterior part of head with dense and short setae. Labrum with a single tooth at anterior margin. Each eye with ca. 70 ommatidia. Aberrant ocellus located near antennal groove (at upper part of groove).

***Antenna*** (Fig. 11A): Short and stout, with rounded joints; length ca. 3 mm; reaching backward to tarsus of leg 2. Lengths of antennomeres 6 > 5 = 4 = 3 = 2 = 1. Antennomere 6 densely setose, sensilla basiconica surrounding apical disc. Last antennomere thickened and flattened, strongly widened apically, axe-shaped. Shape of antennae sexually dimorphic; thickened, widened apically and slightly flattened in male, in female cylindrical. Apical disc with ca. 50 apical cones. No sclerotized ridge between antennal socket and ommatidia.

***Tömösváry’s organ***: Separated from ommatidia, located on a brim between ommatidia and antennal socket, smaller in diameter than an individual ocellus.

***Gnathochilarium***: Ventral surface with setae, other structures typical of the order. Mandibles not dissected.

***Stigmatic plates*** (Fig. 11C): First stigmatic plate subtriangular; apex rounded, broad; straight towards coxa 1.

***Laterotergites***: Laterotergites 1 and 2 narrow, projecting to a sharp tip.

***Collum***: Surface with very long setae in both anterior and posterior margins, setae located in pits.

***Thoracic shield***: Surface as those of tergites, covered with tiny setae; shallow groove with long setae, slightly broad at anterolateral margin.

***Tergites*** (Fig. 2E, F): Quite dull; surface densely setose, easily seen by normal vision; with numerous and short setae, each locating in tiny pits; tips of paratergites of midbody tergites weakly curved, directed posteroventrad.

***Endotergum*** (Figs 13G, H, 14D, E): Posterior margin flat, regular; tip of setae. Inner section (inner area) with a few setiferous tubercles or setae. Middle section (middle area) with a single row of conspicuous, elliptical cuticular impressions; distance between impressions longer than individual diameter. Bristles arranged in two rows, tip of the longest bristles not extended beyond posterior margin or not reaching to posterior margin.

***Anal shield***: Sexually dimorphic, in female weakly bell-shaped, in male strongly bell-shaped. Outer surface pubescent, setae small and very short, similar to those of tergites. Inner surface (underside) covered by setae; with a single locking carina, half as long as length of last laterotergite.

***Legs*** (Fig. 11B): Leg-pairs 1 and 2 without an apical spine. Leg-pair 1 with two or three ventral spines, leg-pair 2 with four ventral spines. Leg-pair 3 with six ventral spines and one apical spine. Leg-pair 4 with 7–9 ventral spines and 1–3 apical spines. Leg-pairs 5–19 with 7–11 ventral spines and 1–3 apical spines. Last two leg-pairs with eight or nine ventral spines and one or two apical spines. In leg 9, femur 1.4×, tarsus 3.5× longer than wide. Length of tarsus > femur > prefemur > coxa > tibia ≥ postfemur. All podomeres densely setose. Coxa large, with dentate ridge marginally (coxal process). Coxal process absent in leg-pairs 1 and 2. Prefemur without teeth. Femur quite short and stout, slightly extended mesally; mesal margin with 7 or 8 conspicuous teeth, long, conical shape.

***Subanal plate*** (Fig. 11F): Trapeziform, undivided; central margin (tip) slightly rounded, narrow; lateral margin slightly concave. Densely setose.

***Male sexual characters*** (Fig. 11D): Gonopore large, covered with a single, undivided, triangular, sclerotized plate.

Anterior telopods (Fig. 12A, B, D): Telopodite with four telopoditomeres; telopoditomeres 3 and 4 clearly divided by a conspicuous suture; all telopoditomeres sparsely setose, except for telopoditomeres 4 without setae. First telopoditomere rectangular, broad, 1.5× longer than wide. Telopoditomere 2 stout. Process of telopoditomere 2 quite short, subequal in length to telopoditomeres 3; visible in posterior view, but partly seen mesally in anterior view; tip curved and well-rounded, directed mesad, close to basal part of telopoditomere 4. Margin towards telopoditomere 3 with a membranous area carrying a sclerotized process (sp); a process conspicuous, but very short, tip quite sharp. Telopoditomere 3 with three crenulated teeth (cr-T), conspicuous. Telopoditomere 4 very short and stout, conspicuous; tip round, directed mesad; with two small, sclerotized spines in posterior side.

***Posterior telopods*** (Fig. 12A, C, D): Telopodite with four telopoditomeres; telopoditomeres 1 and 2 on both sides with sparse setae, except for apical part of immovable finger (process of telopoditomere 2); telopoditomere 3 at base of inner margin with conspicuous setae, but none for outer margin; telopoditomere 4 without setae. First telopoditomere stout and narrow, ca. half as long as telopoditomere 2. Telopoditomere 2 large, immovable finger (process of telopoditomere 2) relatively shorter than movable finger (consisting of telopoditomeres 3 and 4). Immovable finger slender, twice as long as wide, strongly curved, tip directed anteroventrad; at margin with several conspicuous semi-circular rows of sclerotized spots. Margin towards movable finger with two membranous lobes, conspicuous long, triangular, inner lobe bigger and longer than outer one, tip sharp. Telopoditomere 3 very long and slender, tapering apically, curved, thrice as long as telopoditomere 4; with a long and sclerotized spine located on a large, swollen, membranous lobe; posterior part with a row of 11–12 crenulated teeth (cr-T). Telopoditomere 4 slender, 2× longer than wide; at inner margin with a large, conspicuous, swollen, membranous lobe and with two evident sclerotized spines; tip curving mesad.

***Female sexual characters*** (Fig. 11E): Vulva large, covering ca. 2/3 coxa, located at mesal side, extending mesally to basal third of prefemur. Operculum regularly rounded, margin slightly concave, mesal margin not protruding.

##### Distribution and habitats

(Fig. 16). The new species is known from Phetchaburi, Ratchaburi, and Kanchanaburi provinces. All specimens were collected from limestone habitats.

##### Remarks.

At the field collecting site, grey living specimens blended in perfectly with the brownish grey rock or leaf litter, making it difficult to find the animals. All specimens were infested by tiny, engorged, white, phoretic deutonymphs of an unidentified mite. The mite can often be found especially on the ventral part of the body such as antennal sockets and coxae, and could easily be discerned. The distribution of *Z.viridescens* from Dawei, Myanmar (Tavoy, Lower Burma – Moti Ram), is quite close to where the new species is distributed. However, *Z.panhai*sp. nov. differs from it by having a shorter body length ca. 21 mm (vs. longer, ca. 32 mm) and telopoditomeres 3 and 4 of anterior telopod distinctly separated (vs. indistinctly separated).

###### Unconfirmed species recorded for Thailand

#### Zephronia cf. viridescens

Taxon classificationAnimaliaSphaerotheriidaZephroniidae

Attems, 1936

9C1FEFA0-1492-5CE1-B1FA-A4A807B41495

Zephronia viridescens Attems, 1936: 180; Jeekel 2001: 22.
Zephronia cf. viridescens – Wongthamwanich et al. 2012b: 913; Sukteeka and Thanee 2015: 18.

##### Distribution and habitats.

Originally, this species was reported from Tavoy, Lower Burma (Moti Ram) by Attems (1936) (= Dawei, Tenasserim).

##### Remarks.

Although ecological studies by Wongthamwanich et al. (2012b), and Sukteeka and Thanee (2015) have reported ‘Zephroniacf.viridescens’ from northern and northeastern Thailand, these works do not provide clear and unique characters for the species, and the specimens are not available for re-examination. The original description by Attems (1936) clearly stated that one of the diagnostic characters of *Z.viridescens* is its greenish body color. These contrast with the ‘*viridescens*’ material examined by Wongthamwanich et al. (2012b: fig. 4) and Sukteeka and Thanee (2015: fig. 2), which display a distinct brownish body color. Not only can the brown color be used to discriminate *Z.viridescens* from Thai ‘*viridescens*’ material, but the distribution is remarkably different. *Z.viridescens* was originally described from Dawei in Myanmar while ‘*viridescens*’ specimens have been recorded to inhabit the northern and northeastern regions of Thailand. It seems likely that the ‘*viridescens*’ specimens belong to another species and are distinct from all other known species. Therefore, further systematics study based on fresh specimens from northern and northeastern Thailand is necessary in order to confirm the existence of *Z.viridescens* in Thailand. At this moment, we thus exclude this nominal species from the Thai millipedes.

#### Key to the confirmed species of *Zephronia* in Thailand

**Table:** 

1	Entire body grey (Fig. 2E, F). Setae on tergites very long, extending over the posterior margin (Figs 13G, 14D)	***Z.panhai*sp. nov.**
–	Body brown or green or partly green (not grey) (Figs 1, 2A–D). Setae on tergites short, not extending over the posterior margin (Fig. 13A, C, E)	**2**
2	Second coxa in male with conspicuous membranous lobe (Figs 7D, 9D)	**3**
–	Second coxa in male without membranous lobe, inconspicuous (Figs 3D, 5D)	**4**
3	Female vulval operculum regularly rounded, narrow in posterior view (Fig. 7E). Subanal plate subsemicircular, central margin (tip) shallowly concave (Fig. 7F)	***Z.enghoffi*sp. nov.**
–	Female vulval operculum regularly rounded, margin straight and wide (Fig. 9E). Subanal plate trapeziform, with a conspicuous mesal constriction, central margin (tip) strongly concave (Fig. 9F)	***Z.golovatchi*sp. nov.**
4	Process of telopoditomere 2 of anterior telopod long, almost equal in length to the combination of telopoditomeres 3 and 4 (Fig. 6D). Inner section of endoterga with numerous setiferous setae	***Z.phrain* Likhitrakarn & Golovatch, 2021**
–	Process of telopoditomere 2 of anterior telopod short, subequal in length to telopoditomere 3 (Fig. 4B, C). Inner section of endoterga with a few setiferous setae or without setae (Fig. 13A)	**5**
5	Surface of tergites glabrous. Endoterga: tip of the longest bristles extended beyond posterior margin or extending over posterior margin. Female vulva with a large and pointed operculum, conspicuously protruded	***Z.viridisoma* Rosenmejer & Wesener, 2021**
–	Surface of tergites with setae or hairy (Fig. 1C). Endoterga: tip of the longest bristles not extended beyond posterior margin or not extending over posterior margin (Figs 13A, 14A). Operculum of female vulva not pointed, regularly rounded (Fig. 3E)	**6**
6	Body green or partly green (Fig. 1A–D). Tergites with two brown patches locating at almost middle part of anterior half (Fig. 1A, B). Endoterga: posterior margin not flat, ‘rectangle-wavy’ margin (Fig. 14A); middle section with a single row cuticular impressions, conspicuous (Fig. 13B)	***Z.siamensis* Hirst, 1907**
–	Entire body brown. Tergites without color patch on middle part of anterior half, all brown. Endoterga: posterior margin flat; middle section without a row cuticular impressions, inconspicuous	***Z.lannaensis* Likhitrakarn & Golovatch, 2021**

## Discussion

The exploration of the millipede fauna in Thailand has uncovered a hitherto unknown diversity among the genus *Zephronia*. With the three new species described herein, the Thai giant pill-millipede genus *Zephronia* currently contains seven species that promote the number in the genus to 47 species in total. Considering the recorded species of *Zephronia* in Thailand, all can be found in small distribution area, although two of them (*Z.panhai*sp. nov. and *Z.siamensis*) have been shown to have somewhat wider ranges. However, they still occupy less than approximately 300 km^2^ along the mountain ranges in the North and also gulf of Thailand in the East (Fig. 16). This pattern is also marked in *Sphaerobelum* species (*S.aesculus* Rosenmejer & Wesener, 2021 (Rosenmejer et al. 2021)) by its occurrence at 160 km east of the type locality.

**Figure 15.**
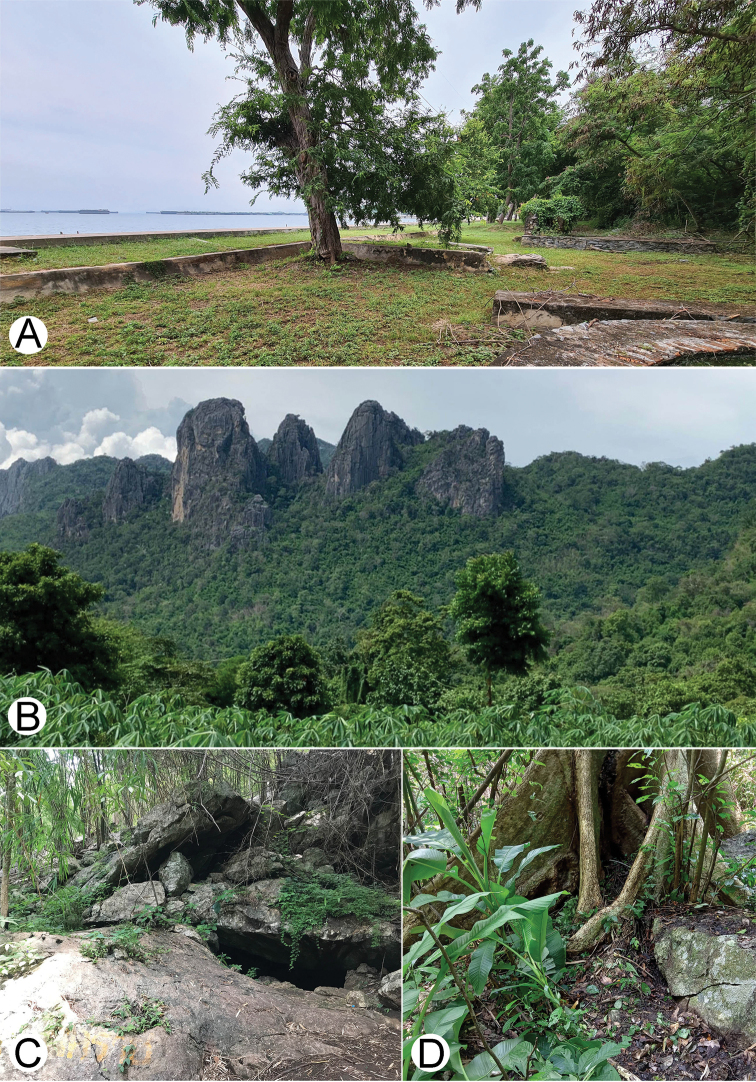
Limestone habitats of some *Zephronia* spp. **A** type locality of *Zephroniasiamensis* (Koh Srichang, Chonburi Province) **B, C** type locality of *Zephroniaenghoffi*sp. nov. (Tham Phaya Nakharat, Khon Kaen Province) **D** Habitat of *Zephroniaphrain* at Phawor shrine, Tak Province.

**Figure 16.**
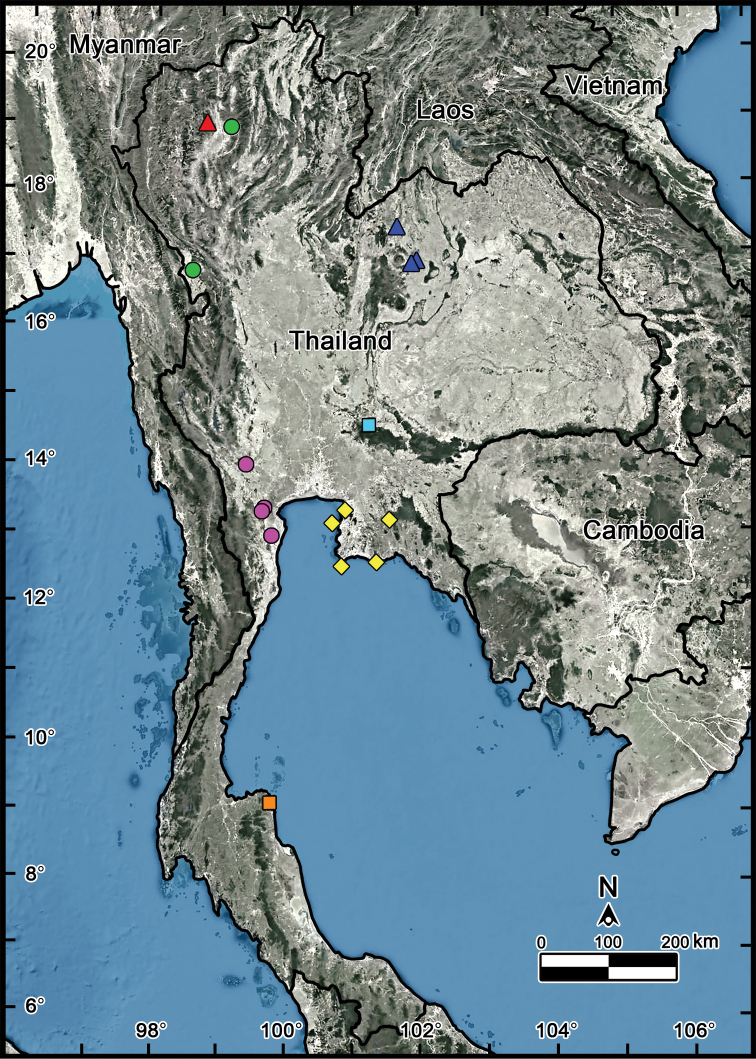
Known distribution of *Zephronia* spp. in Thailand. Red triangle = *Zephronialannaensis*; green circle = *Zephroniaphrain*; blue triangle = *Zephroniaenghoffi*sp. nov.; sky blue square = *Zephroniagolovatchi*sp. nov.; purple circle = *Zephroniapanhai*sp. nov.; yellow diamond = *Zephroniasiamensis* Hirst, 1907; orange square = *Zephroniaviridisoma*.

The species boundaries of *Zephronia* have been mostly based on several morphological features (Wongthamwanich et al. 2012a; Semenyuk et al. 2018; Wesener 2019). The most distinctive characteristics of the three species compared to the congeners can be seen especially in endoterga, anterior telopod and posterior telopod. The use of combinations of characters as being utilities for species discrimination in this study is congruent with previous taxonomic works (Golovatch et al. 2012; Wesener 2016, 2019; Semenyuk et al. 2018, 2020; Likhitrakarn et al. 2021). Furthermore, based on the observations in the field, the live specimens of some species can be easily distinguished from other congeners by their bright body color as presented in *Z.panhai*sp. nov. (Fig. 2E, F), *Z.phrain* (Fig. 1E, F) as well as in *Z.siamensis* (Fig. 1A–D). Based on the combination of several morphological traits plus the live body color, we can confirm that the species boundaries are within *Zephronia*.

As the two recognized groups of *Zephronia* have been proposed based on the location of Tömösváry’s organ (Wesener 2019; Semenyuk et al. 2020; Likhitrakarn et al. 2021), the three new species exhibit all of the unique characters that are in agreement with its placement in *Zephronia* s. s. The group previously harbored nine species, viz., *Z.dawydoffi* Attems, 1953, *Z.konkakinhensis*Semenyuk et al., 2020, *Z.lannaensis* Likhitrakarn & Golovatch, 2021, *Z.montis*Semenyuk et al., 2020, *Z.ovalis* Gray, 1832, *Z.phrain* Likhitrakarn & Golovatch, 2021, *Z.siamensis* Hirst, 1907, *Z.viridescens* Attems, 1936, and *Z.viridisoma* Rosenmejer & Wesener, 2021. Unfortunately, the lack of genetic data prevents a conclusive phylogenetic comparison to other closely related species of the genus at this point. It will be necessary to continue with studies on this group, collecting new material as well as re-examining previously collected material in combination with molecular works. In this way, the systematics within the genus or closely related genera may be elucidated and improved.

The preceding records of the genus, without regarding the three newly described species, were recorded only from northern and eastern parts of Thailand. The central and southern areas of Thailand, which are the intermediate zone between the Malay Peninsula and the upper region of mainland Southeast Asia, had no records of the genus so far. Our finding of these three species fills the gaps in the distribution and confirms the genus *Zephronia* across Thailand. Further collecting in unexplored areas in several parts of mainland Southeast Asia, especially Cambodia, Laos, and Thailand, will probably reveal many new, remarkable species.

## Supplementary Material

XML Treatment for Zephronia

XML Treatment for Zephronia siamensis

XML Treatment for Zephronia lannaensis

XML Treatment for Zephronia phrain

XML Treatment for Zephronia viridisoma

XML Treatment for Zephronia enghoffi

XML Treatment for Zephronia golovatchi

XML Treatment for Zephronia panhai

XML Treatment for Zephronia cf. viridescens

## References

[B1] Attems C (1914) Die Indo-australischen Myriopoden. Archiv für Naturgeschichte 80A (4): 1–398.

[B2] Attems C (1936) Diplopoda of India. Memoirs of the Indian Museum 11: 133–323.

[B3] Attems C (1953) Myriopoden von Indochina, Expedition von Dr. C Dawydoff (1938–1939) Mémoires du Muséum national d’histoire naturelle (n.s., A) 5: 133–230.

[B4] American Veterinary Medical Association (2020) AVMA Guidelines for the Euthanasia of Animals: 2020 edition. https://www.avma.org/sites/default/files/2020-01/2020-Euthanasia-Final-1-17-20.pdf

[B5] Clements R Sodhi NS Schilthuizen M Ng PKL (2006) Limestone karsts of Southeast Asia: Imperiled arks of biodiversity. BioScience 56: 733–742. 10.1641/0006-3568(2006)56[733:LKOSAI]2.0.CO;2

[B6] Decker P (2010) Contributions to the myriapod fauna of Thailand-New records of millipedes and centipedes from Thailand (Myriapoda: Diplopoda, Chilopoda). Schubartiana 4: 23–34. http://www.schubartiana.de/issues/pdf/vol4/Decker-2010-Myriapod_fauna_of_Thailand.pdf

[B7] Enghoff H (2005) The millipedes of Thailand (Diplopoda). Steenstrupia 29(1): 87–103.

[B8] Gray G (1832) The Myriapods (Myriapoda – Mitosata, Fab.). In: Griffith E, Pidgeon E (Eds) The Class Insecta arranged by the Baron Cuvier 1: 124–142.

[B9] Golovatch SI Wesener T Mauriès J-P Semenyuk II (2012) On the identities of *Cryxus* Leach, 1814 and *Zephronia* Gray, 1832, the oldest generic names in the millipede order Sphaerotheriida (Diplopoda). Arthropoda Selecta 21(4): 273–294. 10.15298/arthsel.21.4.01

[B10] Hirst AS (1907) On four new pill-millipedes from the Malay Peninsula and Siam. Annals and Magazine of Natural History (series 7) 20: 215–219. 10.1080/00222930709487327

[B11] Jeekel CAW (2001) A bibliographic catalogue of the Asiatic Sphaerotheriida (Diplopoda). Myriapod Memoranda 3: 5–38.

[B12] Likhitrakarn N Golovatch SI Panha S (2011) Revision of the Southeast Asian millipede genus *Orthomorpha* Bollman, 1893, with the proposal of a new genus (Diplopoda, Polydesmida, Paradoxosomatidae). ZooKeys 131: 1–161. 10.3897/zookeys.131.1921 PMC3208436 22140329

[B13] Likhitrakarn N Golovatch SI Panha S (2014) Three new species of the millipede genus *Tylopus* Jeekel, 1968 from Thailand, with additional notes on species described by Attems (Diplopoda, Polydesmida, Paradoxosomatidae). ZooKeys 435: 63–91. 10.3897/zookeys.435.8286 PMC4141187 25152687

[B14] Likhitrakarn N Golovatch SI Jeratthitikul E Srisonchai R Sutcharit C Panha S (2020) A remarkable new species of the millipede genus *Trachyjulus* Peters, 1864 (Diplopoda, Spirostreptida, Cambalopsidae) from Thailand, based both on morphological and molecular evidence. ZooKeys 925: 55–72. 10.3897/zookeys.925.49953 32317853 PMC7160207

[B15] Likhitrakarn N Golovatch SI Srisonchai R Sutcharit C (2021) Two new species of the giant pill-millipede genus *Zephronia* Gray, 1832 from Thailand (Diplopoda: Sphaerotheriida: Zephroniidae). Tropical Natural History 21(1): 12–26. https://li01.tci-thaijo.org/index.php/tnh/article/view/247730

[B16] Pimvichai P Enghoff H Panha S (2009) A revision of the *Thyropygusallevatus* group. Part 1: the *T.opinatus* subgroup (Diplopoda: Spirostreptida: Harpagophoridae). Zootaxa 1: 17–50. 10.11646/zootaxa.2016.1.2

[B17] Pimvichai P Enghoff H Panha S (2010) The Rhynchoproctinae, a south-east Asiatic subfamily of giant millipedes: cladistic analysis, classification, four new genera and a deviating new species from north-west Thailand (Diplopoda:Spirostreptida:Harpagophoridae). Invertebrate Systematics 24: 51–80. 10.1071/IS09052

[B18] Pimvichai P Enghoff H Panha S Backeljau T (2018) Morphological and mitochondrial DNA data reshuffle the taxonomy of the genera *Atopochetus* Attems, *Litostrophus* Chamberlin and *Tonkinbolus* Verhoeff (Diplopoda: Spirobolida: Pachybolidae), with descriptions of nine new species. Invertebrate Systematics 32: 159–195. 10.1071/IS17052

[B19] Pimvichai P Enghoff H Panha S Backeljau T (2020) Integrative taxonomy of the new millipede genus *Coxobolellus*, gen. nov. (Diplopoda: Spirobolida: Pseudospirobolellidae), with descriptions of ten new species. Invertebrate Systematics 34: 591–617. 10.1071/IS20031

[B20] Pocock RI (1890) On the Myriopoda of Burma. Pt. 1. Report on the Oniscomorpha collected by Sig. L. Fea, by Mr. E.W. Oates and by the late Sig. G.B. Comotto. Annali del Museo Civico di Storia Naturale di Genova 30: 384–395.

[B21] Semenyuk I Golovatch SI Wesener T (2018) Four new species of giant pill-millipedes from Vietnam (Sphaerotheriida, Zephroniidae). Zootaxa 4459(3): 535–550. 10.11646/zootaxa.4459.3.7 30314124

[B22] Semenyuk I Golovatch SI Wesener T (2020) Some new or poorly-known Zephroniidae (Diplopoda, Sphaerotheriida) from Vietnam. ZooKeys 930: 37–60. 10.3897/zookeys.930.47742 32390747 PMC7200893

[B23] Srisonchai R Enghoff H Likhitrakarn N Panha S (2018a) A revision of dragon millipedes I: Genus *Desmoxytes* Chamberlin, 1923, with the description of eight new species (Diplopoda, Polydesmida, Paradoxosomatidae). ZooKeys 761: 1–177. 10.3897/zookeys.761.24214 PMC5988806 29875597

[B24] Srisonchai R Enghoff H Likhitrakarn N Panha S (2018b) A revision of dragon millipedes II: The new genus *Nagaxytes*, with the description of three new species (Diplopoda, Polydesmida, Paradoxosomatidae). European Journal of Taxonomy 462: 1–44. 10.5852/ejt.2018.462

[B25] Srisonchai R Enghoff H Likhitrakarn N Panha S (2018c) A revision of dragon millipedes III: The new genus Gigaxytes, with the description of three new species (Diplopoda, Polydesmida, Paradoxosomatidae). European Journal of Taxonomy 463: 1–43. 10.5852/ejt.2018.463

[B26] Srisonchai R Enghoff H Likhitrakarn N Panha S (2018d) A revision of dragon millipedes IV: The new genus *Spinaxytes*, with the description of nine new species (Diplopoda, Polydesmida, Paradoxosomatidae). ZooKeys 797: 19–69. 10.3897/zookeys.797.29510 PMC6255853 30505161

[B27] Sukteeka S Thanee N (2015) Distribution of Giant Pill-Millipedes (Zephroniacf.viridescens) and Flat-backed millipedes (*Orthomorphavariegata*) in relation to ecological factors at Sakaerat Environmental Research Station, Thailand. International Journal of Advances in Agricultural and Environmental Engineering 2(1): 18–22.

[B28] Wesener T (2016) The giant pill-millipedes, order Sphaerotheriida – An annotated species catalogue with morphological atlas and list of apomorphies (Arthropoda: Diplopoda). Bonn Zoological Bulletin Supplementum 63: 1–104.

[B29] Wesener T (2019) First records of giant pill-millipedes from Laos (Diplopoda, Sphaerotheriida, Zephroniidae). Zootaxa 4563(2): 201–248. 10.11646/zootaxa.4563.2.1 31716539

[B30] Wesener T Sierwald P (2005) The giant pill-millipedes of Madagascar: Revision of the genus *Sphaeromimus*, with a review of the morphological terminology (Diplopoda, Sphaerotheriida, Sphaerotheriidae). Proceedings of the California Academy of Sciences 56(29): 557–599. https://www.biodiversitylibrary.org/page/40743317

[B31] Rosenmejer T Enghoff H Moritz L Wesener T (2021) Integrative description of new giant pill-millipedes from southern Thailand (Diplopoda, Sphaerotheriida, Zephroniidae). European Journal of Taxonomy 762: 108–132. 10.5852/ejt.2021.762.1457

[B32] Wongthamwanich N Panha S Sierwald P Wesener T Thirakhupt K (2012a) A new species of the giant pill-millipede genus *Sphaerobelum* Verhoeff, 1924 from northern Thailand, with an extensive description and molecular characters (Diplopoda: Sphaerotheriida: Zephroniidae). Zootaxa 3220: 29–43. 10.11646/zootaxa.3220.1.2

[B33] Wongthamwanich N Panha S Sitthicharoenchai D Pradatsundarasar A Seelanan T Enghoff H Thirakhupt K (2012b) Daily activities of the giant pill-millipede Zephroniacf.viridescens Attems, 1936 (Diplopoda: Sphaerotheriida: Zephroniidae) in a deciduous forest in Northern Thailand. Zoological Studies 51: 913–926.

